# The enzyme SMPDL3b in podocytes decouples proteinuria from chronic kidney disease progression in experimental Alport Syndrome

**DOI:** 10.1016/j.kint.2025.04.024

**Published:** 2025-05-30

**Authors:** Alla Mitrofanova, Antonio M. Fontanella, Judith Molina, Guanshi Zhang, Shamroop K. Mallela, Luisa Ulloa Severino, Javier Varona Santos J., Matthew Tolerico, Rachel Njeim, Wadih Issa, Maria Boulina, Arianna Carrazco, Veronika Semenova, Yiqin Zuo, Maria Ficarella, Jin Ju Kim, Alexis Sloan, Kumar Sharma, Darren A. Yuen, Laura Perin, George W. Burke, Alessia Fornoni, Sandra Merscher

**Affiliations:** 1 Katz Family Division of Nephrology and Hypertension, Department of Medicine, University of Miami, Miller School of Medicine, Miami, Florida, USA; 2 Peggy and Harold Katz Family Drug Discovery Center, University of Miami, Miller School of Medicine, Miami, Florida, USA; 3 Division of Nephrology, Center for Precision Medicine, Long School of Medicine, University of Texas Health San Antonio, San Antonio, Texas, USA; 4 Division of Nephrology, Department of Medicine, Unity Health Toronto and University of Toronto, Toronto, Canada; 5 Division of Nephrology, American University of Beirut, Beirut, Lebanon; 6 Diabetes Research Institute, University of Miami, Miller School of Medicine, Miami, Florida, USA; 7 Department of Pathology, University of Miami Medical Group, Miller School of Medicine, Miami, Florida, USA; 8 Department of Precision and Regenerative Medicine and Ionian Area (DIMEPRE-J), University of Bari Aldo Moro, Bari, Italy; 9 GOFARR Laboratory, Children’s Hospital Los Angeles, Division of Urology, Saban Research Institute, Keck School of Medicine, University of Southern California, Los Angeles, California, USA; 10 Department of Surgery, Miami Transplant Institute, University of Miami, Miller School of Medicine, Miami, Florida, USA; 11 Department of Microbiology and Immunology, University of Miami, Miller School of Medicine, Miami, Florida, USA

**Keywords:** Alport syndrome, podocyte, proteinuria, SMPDL3b, stiffness

## Abstract

**Background:**

Chronic kidney disease, including Alport Syndrome, is linked to collagen type IV mutations, lipid dysmetabolism, and altered sphingolipid pathways, with no targeted therapies currently available. Sphingomyelin phosphodiesterase acid-like 3b (SMPDL3b), a key regulator of sphingolipid metabolism and membrane receptor organization in podocytes, may drive disease via ceramide and sphingosine-1-phosphate pathways. This study tested whether altered SMPDL3b expression contributes to glomerular injury and renal decline in Alport Syndrome.

**Methods:**

Archived Alport Syndrome human biopsies were used for immunohistochemistry and NanoString re-analysis of SMPDL3b. Murine podocytes isolated from mouse models of Alport Syndrome were profiled using Illumina. Mouse models of Alport Syndrome and models with either podocyte-specific deletion or inducible overexpression of *Smpdl3b* were generated to assess renal function using liquid chromatography–mass spectrometry, matrix-assisted laser desorption ionization–mass spectrometry imaging and atomic force microscopy.

**Results:**

We found a three-fold increase in SMPDL3b expression in glomeruli, tubules and murine podocytes isolated from Col4a3 knockout mice. Increased SMPDL3b expression occurred in association with alterations affecting kidney sphingolipid metabolism, increased glomerular but not tubular sphingosine-1-phosphate levels and reduced glomerular basement membrane and podocyte stiffness. Podocyte-specific *Smpdl3b* deletion in Col4a3 knockout mice was sufficient to restore sphingosine-1-phosphate levels, to reduce proteinuria, podocyte foot process effacement, and improve glomerular basement membrane and podocyte stiffness, but not sufficient to protect from kidney failure.

**Conclusions:**

Our study suggests that SMPDL3b may be a key modulator of proteinuria and podocyte integrity in Alport Syndrome, decoupling proteinuria from kidney failure, and suggests that improvement of glomerular structure and function may not always translate into protection from chronic kidney disease progression.

Chronic kidney disease (CKD) is a major health care problem worldwide,^1^ which is expected to become a leading cause of death globally in the near future.^2^ Recent genome-wide association studies discovered many genetic mutations associated with CKD, kidney function, and albuminuria,^3,4^ and collagen type IV mutations are among the most prevalent mutations in patients with CKD.^5–7^ More recently, the presence of Col4 variants in 2 very large patient datasets was found to increase the genome-wide polygenic scores for CKD.^8^ In turn, mutations in the genes coding for different alpha chains of collagen type IV (*Col4a3*, *Col4a4*, *Col4a5*) are also characteristic for patients with Alport syndrome (AS), who present with a renal phenotype characterized by disruption of the glomerular filtration barrier, hematuria, proteinuria, and progressive glomerular and tubulointerstitial fibrosis, ultimately leading to renal failure^9^ among other nonrenal manifestations. Some patients with AS develop kidney failure during adolescence or early childhood, nevertheless no specific treatment for AS is available, and ramipril represent the only standard of care based on retrospective and prospective studies.^10,11^

We previously reported that Col4a3 knockout (KO) mice (Col4a3^−/−^), a mouse model of autosomal recessive AS,^12^ are characterized by accumulation of cholesterol esters, cholesterol crystals, and triglycerides^13^ and have increased numbers of glomerular lipid droplets *in vivo* and podocyte lipid droplets *in vitro.*^14,15^ Similarly, others reported lipid accumulation in tubules of Col4a3^−/−^ mice.^16^ Our recent studies demonstrated that inhibition of triglyceride accumulation via ezetimibe,^14^ as well as induction of cholesterol efflux with a compound that is currently being tested in phase II clinical trial for patients with AS,^17^ may both be valuable strategies to slow disease progression in AS. These observations underline the importance of lipid dysmetabolism as a contributor to the progression of AS. However, whether sphingolipid metabolism contributes to CKD progression in AS remains unclear.

Sphingolipids belong to a diverse class of lipids with their most critical role being components of lipid rafts, the sphingomyelin- and cholesterol-rich microdomains of the plasma membrane, where they regulate intracellular signal transduction.^18^ Ceramide is the centerpiece of sphingolipid metabolism. Briefly, ceramide is produced either by *de novo* synthesis or by turnover of other sphingolipids. *De novo* synthesis of ceramide occurs at the endoplasmic reticulum from serine and palmitoyl-CoA by ceramide synthase. Ceramide can also be generated from sphingomyelin by the action of sphingomyelinases. Ceramide accumulation is toxic to a cell and thus, ceramide is converted into ceramide-1-phosphate (C1P) by CERK or sphingosine-1-phosphate (S1P) by sphingosine kinases (SPHK1/SPHK2),^19^ the bioactive sphingolipids that regulate cell survival and inflammatory responses in cells.

S1P is involved in many physiological processes and contributes to the pathogenesis of many diseases as reviewed elsewhere.^20^ The association of S1P with multiple diseases results, in part, from its action as an intracellular (as a second messenger in the intracellular compartments) and extracellular (through S1PR1–S1PR5) signaling molecule. Intracellular versus extracellular S1P signaling may have differing effects depending on the renal cell type and may contribute differently to specific kidney diseases.^21^ Interestingly, mutations in *SGPL1*, the gene that encodes S1P lyase and irreversibly degrades S1P into hexadecenal and ethanolamine-1-phosphate, have been reported to be associated with steroid-resistant nephrotic syndrome.^22–25^ However, research investigating the contribution of sphingolipids, and particularly S1P signaling, to the progression of renal disease in AS is sparse. A recent study reported an increase in 2 specific sulfatide species—SulfoHex-Cer (d18:2/24:0) and SulfoHex-Cer (d18:2/16:0)—in kidney cortices from Col4a3^−/−^ mice during development,^26^ suggesting that sphingolipids may contribute to altered glomerular development and renal failure in AS. Interestingly, cross-talk between cholesterol and sphingolipid metabolism has been recently shown, where activation of farnesoid X receptor suppresses renal SPHK1 expression and inhibits S1P signaling, which results in reduced neutrophilic inflammation and neutrophil-specialized programmed cell death in male but not female Col4a3^−/−^ mice.^27^ The same study demonstrated that kidney S1P levels in Col4a3^−/−^ mice are 2 times higher compared to CTRL mice. Similarly, we described an important role for sphingolipids in glomerular diseases^18,28,29^ and demonstrated that SMPDL3B, an enzyme expressed in lipid raft domains in podocytes, regulates the assembly of plasma membrane receptors, such as of the insulin receptor in caveolin-rich plasma membrane domains, activates αγβ3 integrin activation and migration, and regulates the availability of C1P.^18,30^ In macrophages, SMPDL3B was shown to regulate plasma membrane fluidity by affecting the plasma membrane sphingolipid and ceramide content.^31^

The overall goal of the present study was to determine whether altered SMPDL3B expression affects the generation of S1P, thereby contributing to glomerular injury, proteinuria, and renal failure in AS.

Col4a3^−/−^ mice, mice with doxycycline-induced podocyte-specific *Smpdl3b* overexpression and podocyte *Smpdl3b*-deficient Col4a3^−/−^ mice were utilized to investigate how altered SMPDL3B expression affects sphingolipid metabolism in the kidney and, particularly, in glomeruli and whether targeting SMPDL3B may represent a novel therapeutic strategy to protect from experimental AS. Here we demonstrate that Col4a3^−/−^ mice are characterized by increased glomerular SMPDL3B expression, which is associated with increased renal cortical S1P content and decreased glomerular basement membrane (GBM) and podocyte stiffness. Overexpression of SMPDL3B in podocytes isolated from Col4a3^−/−^ mice and in mice with doxycycline-inducible podocyte-specific *Smpdl3b* overexpression is associated with increased renal cortical S1P and decreased C1P levels. Podocyte-specific *Smpdl3b* deletion in Col4a3^−/−^ mice is sufficient to reduce proteinuria and podocyte foot process effacement, but surprisingly did not protect from podocyte loss and renal failure. Decreased SMPDL3B expression in podocytes also results in reduced levels of S1P in kidney cortex and glomeruli. Exogenous S1P administration worsens proteinuria and leads to reduced synaptopodin expression in association with increased apoptosis in podocyte *Smpdl3b*-deficient Col4a3^−/−^ mice and to increased apoptosis in murine wild-type (control) and AS podocytes *in vitro*.

Taken together, our findings demonstrate that sphingolipid metabolism is altered in AS, and they shed light on a novel mechanism in which SMPDL3B modulates S1P signaling, podocyte rigidity, and proteinuria in AS but does not affect renal failure progression. Because proteinuria is among the major determinants of disease progression in AS, our study challenges the idea that improvement of proteinuria and glomerular structure via modulation of SMPDL3B-derived S1P translates in protection from CKDprogression. Our study also reveals for the first time that increased production of biologically active lipids, such as S1P, may contribute to worsening proteinuria.

## METHODS

### Study approval

All animal studies complied with the relevant ethical regulations and were performed in accordance with guidelines from the National Institutes of Health, Animal Research: Reporting of In Vivo Experiments (ARRIVE), and TRAnslational Nephrology Science FOR new Medications (TRANSFORM). The study protocol was approved by the Institutional Animal Care and Use Committee of the University of Miami, Miller School of Medicine.

### Human kidney biopsy

Archived biopsy samples from patients with AS stored at Children’s Hospital Los Angeles, as previously published,^32^ were used for immunohistochemistry only (see the Supplementary Extended Methods). Publicly available data processed for NanoString GeoMx DSP (digital spatial profiling) from these samples (GSE255265) were reanalyzed to detect *SMPDL3B* expression.

### Generation of Col4a3 KO mice with podocyte specific *Smpdl3b* deficiency

Col4a3 heterozygous (Col4a3^+/−^) mice were purchased in a 129X1/SvJ background (129-Col4a3^tm1Dec^/J, #002908, Jackson Laboratories) and backcrossed to C57BL6/J mice (#000664, Jackson Laboratories) for 10 generations. Then, Col4a3^+/−^ littermates were bred to generate homozygous Col4a3 KO mice in a C57BL6 background (Col4a3^−/−^). Col4a3^−/−^ mice were bred to homozygous podocyte-specific *Smpdl3b*-deficient mice (Smp^−/−^)^18^ to obtain double heterozygous mice. Finally, the latter were intercrossed to generate homozygous Col4a3^−/−^ mice with podocyte-specific homozygous *Smpdl3b* deficiency (DKO). Wild-type littermates (CTRL) and Smp^−/−^ mice were used as controls. A detailed scheme for the generation of DKO mice is shown in Supplementary Figure S1A. Experimental animals of both sexes were 4 and 20 weeks old.

### Generation of mice with doxycycline-inducible podocyte-specific *Smpdl3b* overexpression

To generate mice with a doxycycline-inducible podocyte-specific *Smpdl3b* overexpression, a Myc-DDK-SMPDL3B cDNA was utilized to amplify *Smpdl3b* using specific primers (Supplementary Table S1) followed by separation using gel electrophoresis. The band corresponding to the full-length cDNA of *Smpdl3b* was excised, blunted, and purified using the Gel Extraction and PCR Purification kits (Qiagen) and then cloned into the pTRE3G vector, which contains an inducible Tet-responsive element. To generate an Smpdl3b inducible vector Myc-DDK-SMPDL3b-pTRE3G, the blunted cDNA product was cloned into a blunted vector and TetO-Myc-DDK-SMPDL3B insert was released from the vector backbone using Ase I and utilized for pronuclear injection in fertilized oocytes of C57BL/6 mice to generate *Smpdl3b* transgenic mice (SMP^Tg^). To generate mice with podocyte-specific doxycycline inducible Myc-DDK-SMPDL3B overexpression (pSMP^Tg^), SMP^Tg^ founders were bred to podocinrtTA mice^33^ (Supplementary Figure S2A). Podocyte SMPDL3B expression was induced by feeding the mice 2000–parts per million doxycycline-containing (Dox+) chow for 1 or 16 months, starting at 7 weeks of age. Experimental animals of both sexes were 2 and 16 months old.

### S1P response experiments

*In vivo*, 16-week-old mice were treated with 0.0002 mg/kg S1P (d17:1) or 5% dimethyl sulfoxide (DMSO) (i.p. injections, in 4 mg/ml fatty acid–free albumin solution) daily for 4 weeks. Spot morning urines and body weight measurements were collected weekly. Blood samples and kidneys were collected at sacrifice time point and processed for indepth phenotypical analysis as described in Supplementary Extended Methods. *In vitro*, murine podocytes isolated from Col4a3^−/−^ mice^34^ were treated with 100 nmol/l S1P (d17:1) at concentrations 0.1, 0.2, 1 and 5 μmol/l at 37 °C in fetal bovine serum–free Dulbecco’s modified Eagle’s medium/F12 media for 24 hours.

### Animal housing

For all *in vivo* experiments, mice were housed under a 12:12 hour light-dark cycle in standard rodent cages under controlled temperature (22 ± 1 °C) and provided water and Teklad Global 18% protein rodent chow diet (Alice) *ad libitum*. Animals of both sexes—male and female—were used. No inclusion or exclusion criteria were used to assign animals into groups. For genotyping details, groups assignment, and histologic analyses used, see the Supplementary Extended Methods.

### Immunoblotting

See the Supplementary Extended Methods. Antibodies are listed in Supplementary Table S2.

### RNA extraction and quantitative reverse transcriptase polymerase chain reaction

See the Supplementary Extended Methods. Primer sequences are listed in Supplementary Table S1.

### Illumina bulk RNA-sequencing analysis

See the Supplementary Extended Methods. Preparation and sequencing of RNA libraries was carried out in the John P. Hussman Institute for Human Genomics Center for Genome Technology, University of Miami.

### Immunohistochemistry

See the Supplementary Extended Methods. Antibodies are listed in Supplementary Table S2.

### Liquid chromatography–mass spectrometry analysis

Kidney tissue homogenates (at least 25 mg), cultured murine podocytes (at least 1 × 10^6^ cells per sample) or urine collected from mice (50–200 μl) were used. Cell pellets and kidney tissue were subjected to liquid extraction as reported previously.^35^ For more details, see the Supplementary Extended Methods.

### Matrix-assisted laser desorption ionization–mass spectrometry imaging

See the Supplementary Extended Methods. A multimodal imaging approach, including bright-field and autofluorescence microscopy, was employed to visualize glomerular morphology, with periodic acid–Schiff staining used to validate glomerular localization. Lipid molecule imaging was conducted in both negative and positive ion modes using specific matrix solutions and optimized spraying parameters, while mass spectrometry imaging data were acquired with a Q Exactive HF-X mass spectrometer (Thermo Fisher Scientific).

### Atomic force microscopy force spectroscopy

Podocytes, GBM, and glomerular stiffness were evaluated in frozen kidney sections using a slightly modified version of the previously published protocol.^36^ See the Supplementary Extended Methods.

### Statistical analysis and study design

Data are expressed as a mean ± SD. Several experiments ranging between 3 and 5 were utilized and as indicated for each distinct experiment. No randomization was used. Investigators were blinded for imaging and data analyses. Between-group differences were measured using a 2-tailed Student’s *t* test or 1-way analysis of variance with *post hoc* Tukey’s analysis where appropriate. Statistical analysis was performed using GraphPad Prism 10.0 (Dotmatics). *P* < 0.05 was considered significant. For sample size calculations and statistics used for matrix-assisted laser desorption ionization–mass spectrometry imaging, see the Supplementary Extended Methods.

## RESULTS

### Increased SMPDL3B expression in clinical and experimental AS is associated with altered sphingolipid content

**Figure 1 |:**
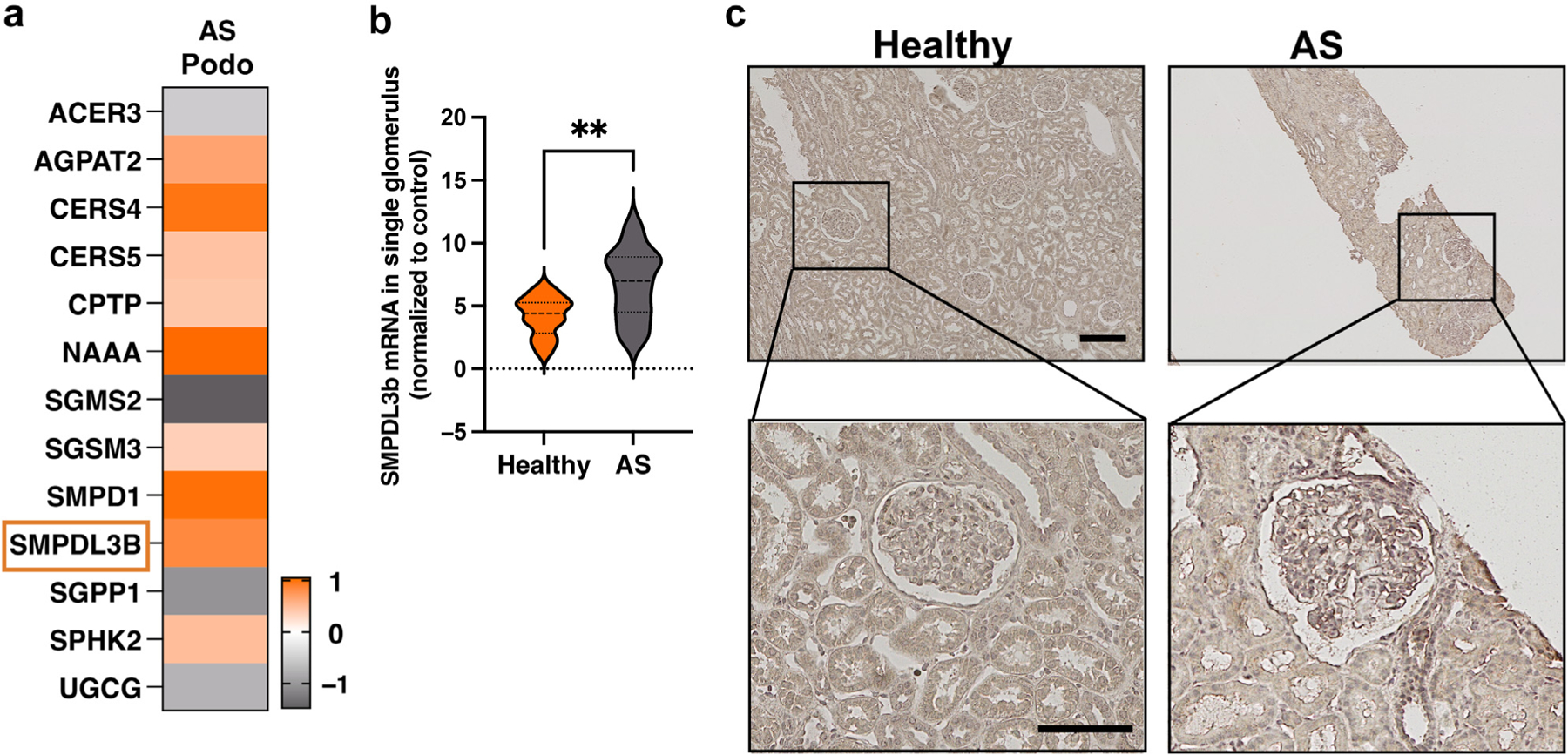
SMPDL3B expression is elevated in patients with Alport syndrome (AS). (**a**) Bulk RNA-sequencing analysis of urine-derived podocytes (Podo) from 3 patients with AS (GSE134011). Genes that passed false discovery rate correction (*q* ≤ 0.05) for multiple testing were considered as significantly regulated and are highlighted in *orange* (increased expression) or *gray* (decreased expression). (**b**) Violin plot showing the digital spatial profiling analysis of glomerular SMPDL3B expression from patients with AS (n = 9 females and n = 8 males) (GSE255265). (**c**) Representative immunoperoxidase staining of kidney sections (4 μm) for SMPDL3B in healthy controls and patients with AS. Original magnification ×20; bar = 30 μm. **P < 0.01. To optimize viewing of this image, please see the online version of this article at www.kidney-international.org.

**Figure 2 |:**
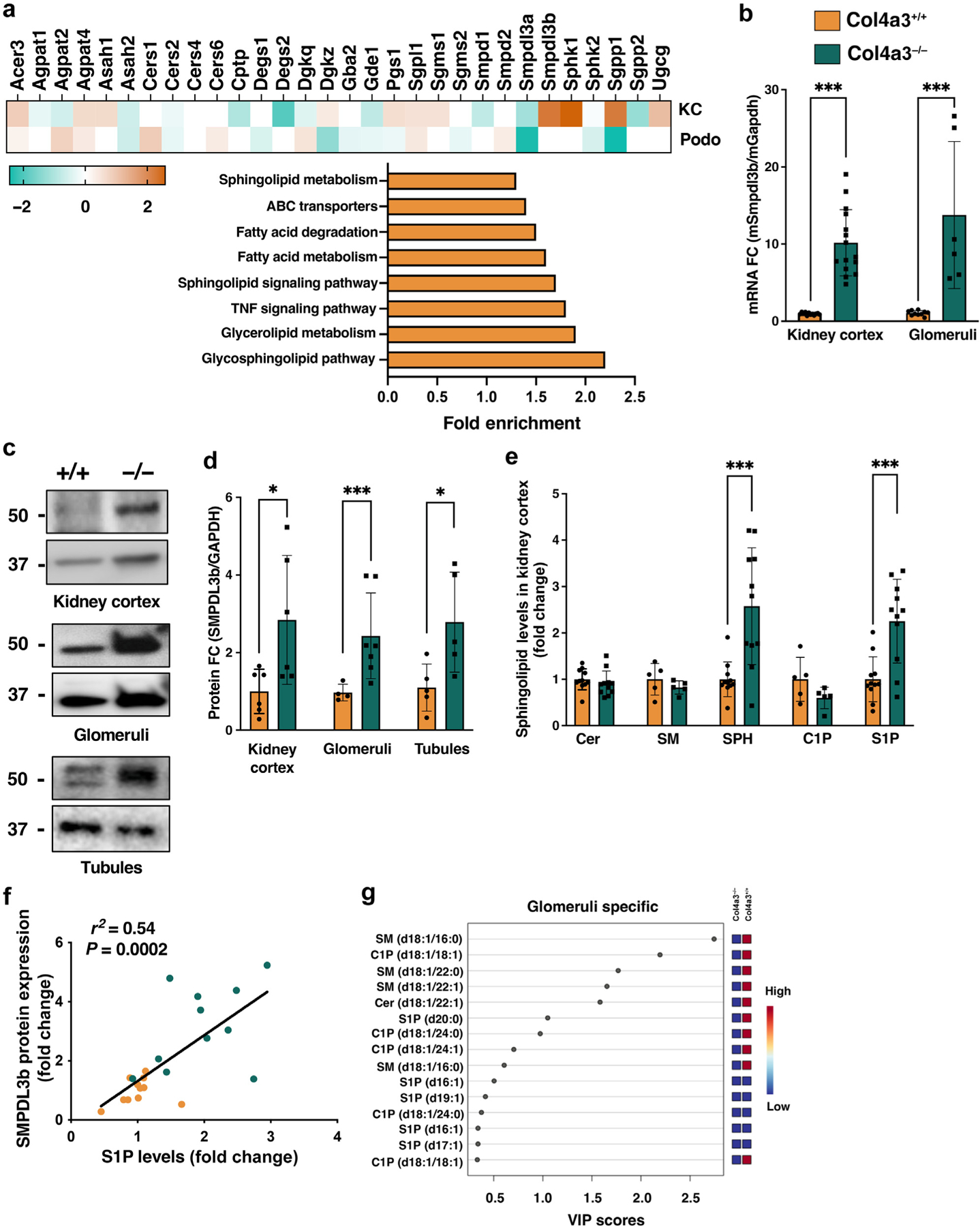
Increased glomerular SMPDL3B expression in Col4a3^−/−^ mice is associated with dysregulated sphingolipid metabolism. (**a**) Bulk RNA-sequencing analysis of kidney cortices (KC) isolated from Col4a3^−/−^ mice (n = 3) and immortalized murine podocytes (Podo) isolated from 8-week-old Col4a3^−/−^ mice. Genes that passed false discovery rate correction (*q* ≤ 0.05) for multiple testing were considered as significantly regulated and are highlighted in *orange* (increased expression) or *green* (decreased expression). Insignificantly detectable or undetectable genes are highlighted in *white*. A gene set enrichment analysis showing the fold enrichment for 8 relevant pathways based on the list of significantly changed genes. (**b**) mRNA expression levels of *Smpdl3b* in KC and glomeruli isolated from wild-type (Col4a3^+/+^) and Col4a3^−/−^ mice. Analysis is done using 15 mice per group for KC and 6 per group for pooled glomeruli. Representative Western blot (**c**) and bar graphs (**d**) of SMPDL3B expression in KC, glomeruli, and tubules isolated from Col4a3^+/+^ (+/+) and Col4a3^−/−^ (−/−) mice. (**e**) Liquid chromatography–mass spectrometry analysis of total ceramide (Cer), sphingomyelin (SM), sphingosine (SPH), ceramide-1-phosphate (C1P), and sphingosine-1-phosphate (S1P) levels in KC isolated from Col4a3^+/+^ and Col4a3^−/−^ mice (n = 5–11 mice per group). (**f**) Correlation analysis between SMPDL3B mRNA expression and S1P levels in KC isolated from Col4a3^+/+^ (*orange dots*) and Col4a3^−/−^ (*green dots*). Linear regression used with *r*^*2*^ and *P* values shown. (**g**) Associated Variable Importance in Projection (VIP) scores ranking the most variable levels of SM, S1P, and C1P in glomeruli region from Col4a3^+/+^ and Col4a3^−/−^ mice using matrix-assisted laser desorption ionization–mass spectrometry imaging assay. Data are mean ± SD. *P < 0.05; ***P < 0.001. *P* values were calculated using 2-tailed Student’s *t* test (**b–e**). ABC, adenosine triphosphate binding cassette; TNF, tumor necrosis factor.

We previously described SMPDL3B as an enzyme expressed in podocyte lipid rafts that might play an important role in the pathogenesis of proteinuric kidney diseases^28,29^ and might regulate the availability of bioactive sphingolipids such as C1P^18^ and S1P.^37^ Using whole-transcript array data obtained from urine-derived podocytes from patients with AS (GSE134011), we confirmed increased *SMPDL3B* expression in association with altered mRNA expression of other genes that regulate sphingolipid metabolism (Figure 1a). In further support, digital spatial profiling analysis revealed increased glomerular SMDPL3B expression in kidney biopsies from patients with AS (GSE255265) (Figure 1b), which was further confirmed using immunoperoxidase staining (Figure 1c). To determine whether SMPDL3B is differentially expressed in Col4a3^−/−^ mice, an experimental model of AS, we also performed an analysis of openly available whole-transcript array data of kidneys from 8-week-old Col4a3^−/−^ mice (GSE109777). We found dysregulated expression of several sphingolipid genes in kidney cortices of Col4a3^−/−^ mice, where SMPDL3B expression was upregulated (Figure 2a). Transcriptional analysis using immortalized murine podocytes isolated from Col4a3^−/−^ mice (IMAS) revealed 6502 differentially expressed genes (GSE274298) (Supplementary Figure S3A), with a substantial portion of these genes involved in sphingolipid and fatty acid metabolism (Figure 2a and Supplementary Data 1). Quantitative reverse transcriptase polymerase chain reaction analysis confirmed significantly increased *Smpdl3b* mRNA expression in kidney cortex and glomeruli isolated from Col4a3^−/−^ mice (Figure 2b), as well as in IMAS podocytes (Supplementary Figure S3B). Interestingly, *Smpdl3b* mRNA expression in glomeruli from Col4a3^−/−^ mice was significantly increased starting at 4 weeks of age and continued to gradually increase with age and renal disease progression (Supplementary Figure S3C). Importantly, protein expression of SMPDL3B was significantly increased in kidney cortex, glomeruli, and tubules fraction isolated from 8-week-old Col4a3^−/−^ mice (Figure 2c and d) and IMAS podocytes (Supplementary Figure S3D and E). To determine whether increased SMPDL3B expression is associated with changes in the renal content of sphingolipids, liquid chromatography–mass spectrometry analysis of kidney cortices isolated from wild-type (Col4a3^+/+^) and Col4a3^−/−^ mice was performed. While total ceramide, total sphingomyelin, and total C1P levels remained unchanged, total sphingosine and total S1P levels were significantly elevated in Col4a3^−/−^ mice (Figure 2e). Interestingly, while changes in the total C1P content were not significant between the groups, C18:1, C20:0, and C24:0 C1P species were significantly reduced in Col4a3^−/−^ mice compared to controls (Supplementary Data 2). In addition, total C1P levels in IMAS podocytes were also reduced (Supplementary Figure S3F and Supplementary Data 3), which is consistent with our previously published data^18^ and supports the hypothesis that SMPDL3B alters the availability of bioactive sphingolipids. In further support, renal SMPDL3B expression significantly correlated with S1P levels in Col4a3^−/−^ mice (Figure 2f), increased glomerular S1P (Figure 2g and Supplementary Figure S4A), and decreased C1P levels as revealed using matrix-assisted laser desorption ionization–mass spectrometry imaging analysis (Figure 2g and Supplementary Figure S4A and B). The evidence that elevated renal expression of SMPDL3B correlates with increases in renal S1P levels in Col4a3^−/−^ mice prompted us to investigate whether podocyte-specific *Smpdl3b* deficiency would be sufficient to restore renal S1P content and protect from CKD progression.

### Podocyte-specific *Smpdl3b* deficiency in Col4a3^−/−^ mice protects from albuminuria and foot processes effacement but not from glomerulosclerosis and loss of renal function

**Figure 3 |:**
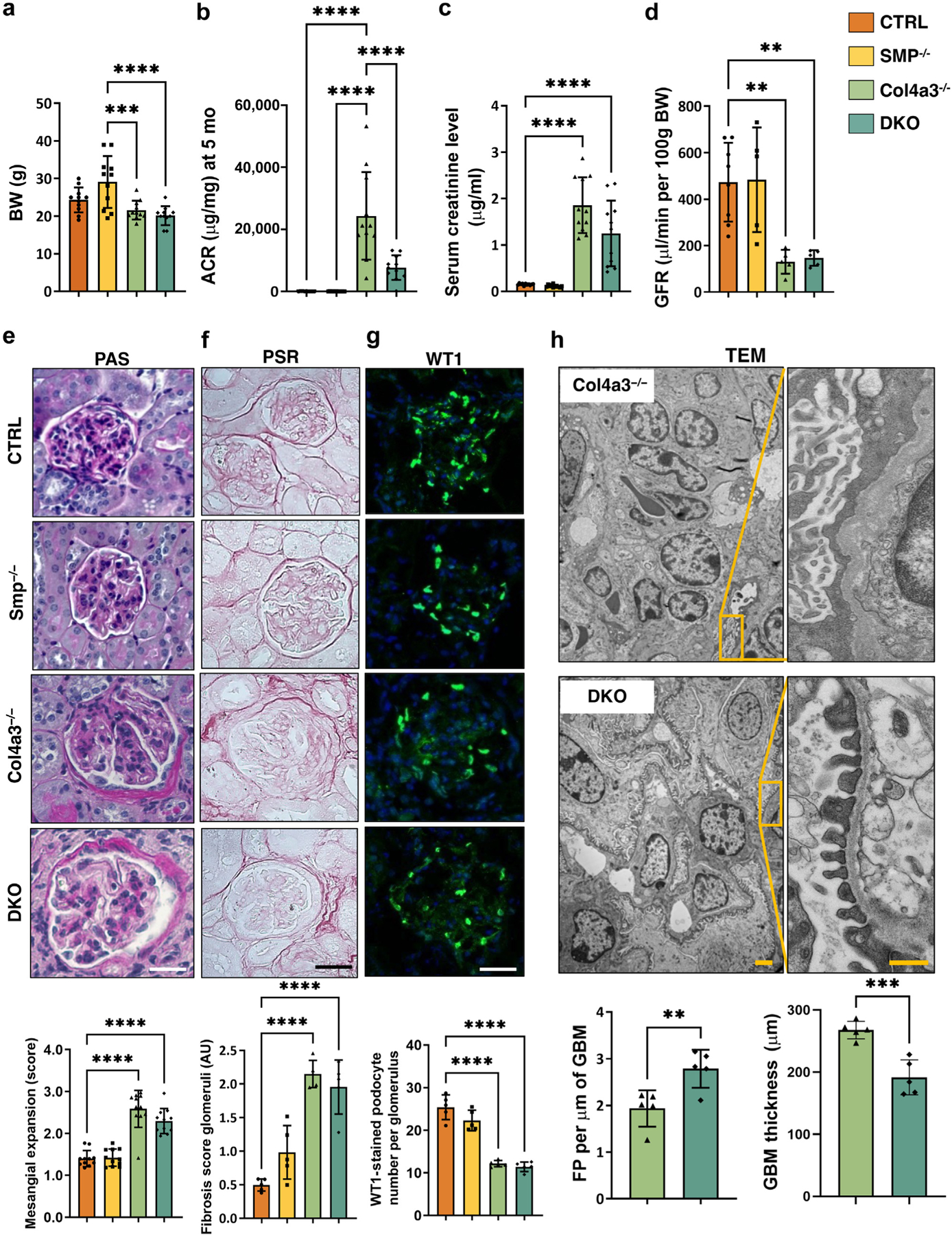
Renal phenotype of Col4a3^−/−^ with podocyte-specific *Smpdl3b* deficiency (DKO). Four experimental groups of mice were used in the study: (i) wild-type control mice (CTRL, n = 11); (ii) mice with podocyte-specific *Smpdl3b* deficiency (Smp^−/−^,n = 11); (iii) Col4a3^−/−^ mice (n = 11); and (iv) Col4a3−/− mice with podocyte-specific *Smpdl3b* deficiency (DKO, n = 11). Body weight (BW) (**a**), albumin–creatinine ratio (ACR) (**b**), serum creatinine level (**c**), and glomerular filtration rate (GFR) (**d**) in all 4 experimental groups of mice. Representative periodic acid–Schiff (PAS) staining (**e**), picrosirius red (PSR) staining (**f**), and immunostaining for anti-Wilms’ tumor 41 (WT1, green) (**g**) of kidney sections (4 μm) and bar graph analysis (bottom panel) in all 4 groups of mice. Original magnification ×40; bar = 30 μm. (**h**) Representative semi-thin and scaled transmission electron microscopy (TEM) images and bar graph analysis of foot process (FP) effacement and glomerular basement membrane (GBM) thickness (bottom panel) in Col4a3^−/−^ and DKO mice. Original magnification ×30,000; bar = 4 μm and 500 nm for semi-thin and scaled images, respectively. Data are mean ± SD. **P < 0.01; ***P < 0.001; ****P < 0.0001. *P* values were calculated using 1-way analysis of variance (**a–g**) and 2-tailed Student’s *t* test (**h**). AU, arbitrary units. To optimize viewing of this image, please see the online version of this article at www.kidney-international.org.

We next generated Col4a3^−/−^ mice with podocyte-specific *Smpdl3b*-deficient (DKO) mice (see the Supplementary Extended Methods and Supplementary Figure S1). We analyzed *Smpdl3b* expression levels in glomeruli isolated from 4 experimental groups (CTRL mice, podocyte-specific *Smpdl3b-*deficient [Smp^−/−^] mice, Col4a3^−/−^ mice, and DKO mice). As expected, Smpdl3b expression was significantly decreased on mRNA (Supplementary Figure S1C) and protein (Supplementary Figure S1D and, E) levels in Smp^−/−^ and DKO mice, while *Smpdl3b* expression levels were significantly increased in Col4a3^−/−^ mice when compared to CTRL mice. While Col4a3^−/−^ mice on a 129-background die of renal failure around 8 weeks of age, Col4a3^−/−^ mice on C57Bl/6 background and DKO mice are fertile and die between 5 and 6 months of age (Supplementary Figure S5A). At 20 weeks of age, Col4a3^−/−^ and DKO mice had significantly lower body weight compared to CTRL mice with no changes between Col4a3^−/−^ and DKO mice (Figure 3a and Supplementary Figure S5B). Importantly, albumin–creatinine ratio (ACR) in Col4a3^−/−^ mice was significantly elevated compared to CTRL mice but podocyte-specific *Smpdl3b* deficiency significantly reduced ACRs in DKO to levels (Figure 3b and Supplementary Figure S5C), while the ACR remained significantly higher compared to CTRL mice. Interestingly, reduced podocyte *Smpdl3b* expression and ACR improvement in DKO mice did not protect from CKD progression, because serum creatinine (Figure 3c) and the glomerular filtration rate (Figure 3d and Supplementary Figure S5D) were similar in Col4a3^−/−^ and DKO mice and significantly regulated when compared to CTRL mice. Similarly, histologic analysis revealed significantly increased mesangial expansion scores (Figure 3e), glomerulosclerosis (Figure 3f), and tubulointerstitial fibrosis (Supplementary Figure S5E) in Col4a3^−/−^ and DKO mice when compared to CTRL mice. Interestingly, the improvement in the ACR observed in DKO mice when compared to Col4a3^−/−^ mice was also not associated with a preservation of podocyte numbers (Figure 3g), although transmission electron microscopy analysis demonstrated preserved number of foot processes and reduced GBM thickness in DKO compared to Col4a3^−/−^ mice (Figure 3h). Taken together, these results suggest that podocyte-specific *Smpdl3b* deficiency in Col4a3^−/−^ mice improves ACR and foot process effacement but is not sufficient to improve glomerulosclerosis and prevent renal failure, suggesting that podocyte SMPDL3B expression dissociates proteinuria from CKD progression in this model.

The podocyte depletion paradigm suggests that a decrease in podocyte numbers or density below a certain threshold (>40%) underlies the onset of proteinuria and leads irreversibly to the development of global glomerulosclerosis at early stages of CKD progression.^38,39^ Therefore, we hypothesized that absence of histologic or renal function improvements in 5-month-old DKO mice is the result of irreversible podocyte loss at early stages. To test this hypothesis, we analyzed the same 4 groups of mice (CTRL, SMP^−/−^, Col4a3^−/−^, and DKO) at 4 weeks of age. However, mesangial expansion (Supplementary Figure S6A) as well as podocyte number (Supplementary Figure S6B) was similar in all 4 groups, suggesting that a decrease in podocyte number was not the driver of proteinuria in Col4a3^−/−^ and DKO mice, which were already proteinuric at 4 weeks of age (Supplementary Figure S6C).

### SMPDL3B expression affects the availability of bioactive sphingolipids in the kidneys and in urine of mice with AS

Giving the complexity of sphingolipid metabolism, levels of S1P inside a cell are regulated by multiple enzymes, which balance its synthesis from sphingosine, SPHK1, and SPHK2) and its degradation (S1P phosphatases or S1P lyase) directly. Additionally, changes in activity of other enzymes, which convert C1P into ceramide (C1PP/SMPDL3B) and ceramide into sphingosine (ceramidases), or the activity of S1P transporters (SPNS2) indirectly contribute to the regulation of intracellular S1P levels. Therefore, we next investigated expression of genes involved in regulating S1P levels and/or S1P signaling in the kidney cortices of Col4a3^−/−^ mice. However, quantitative reverse transcriptase polymerase chain reaction analysis showed no significant changes in mRNA expression of any S1P signaling-related genes in either 5-month- (Supplementary Figure S5F) or 4-week-old mice (Supplementary Figure S6D). Significant mRNA upregulation of alkaline ceramidase 1 (Acer1) and Spns2 in 5-month-old and Acer1 in 4-week-old Col4a3^−/−^ mice only was found. Podocyte-specific deletion of *Smpdl3b* resulted in increased Acer1 and Spns2 mRNA levels in 4-week-old DKO mice but decreased Acer1 and Spns2 mRNA levels in 5-month-old DKO mice.

**Figure 4 |:**
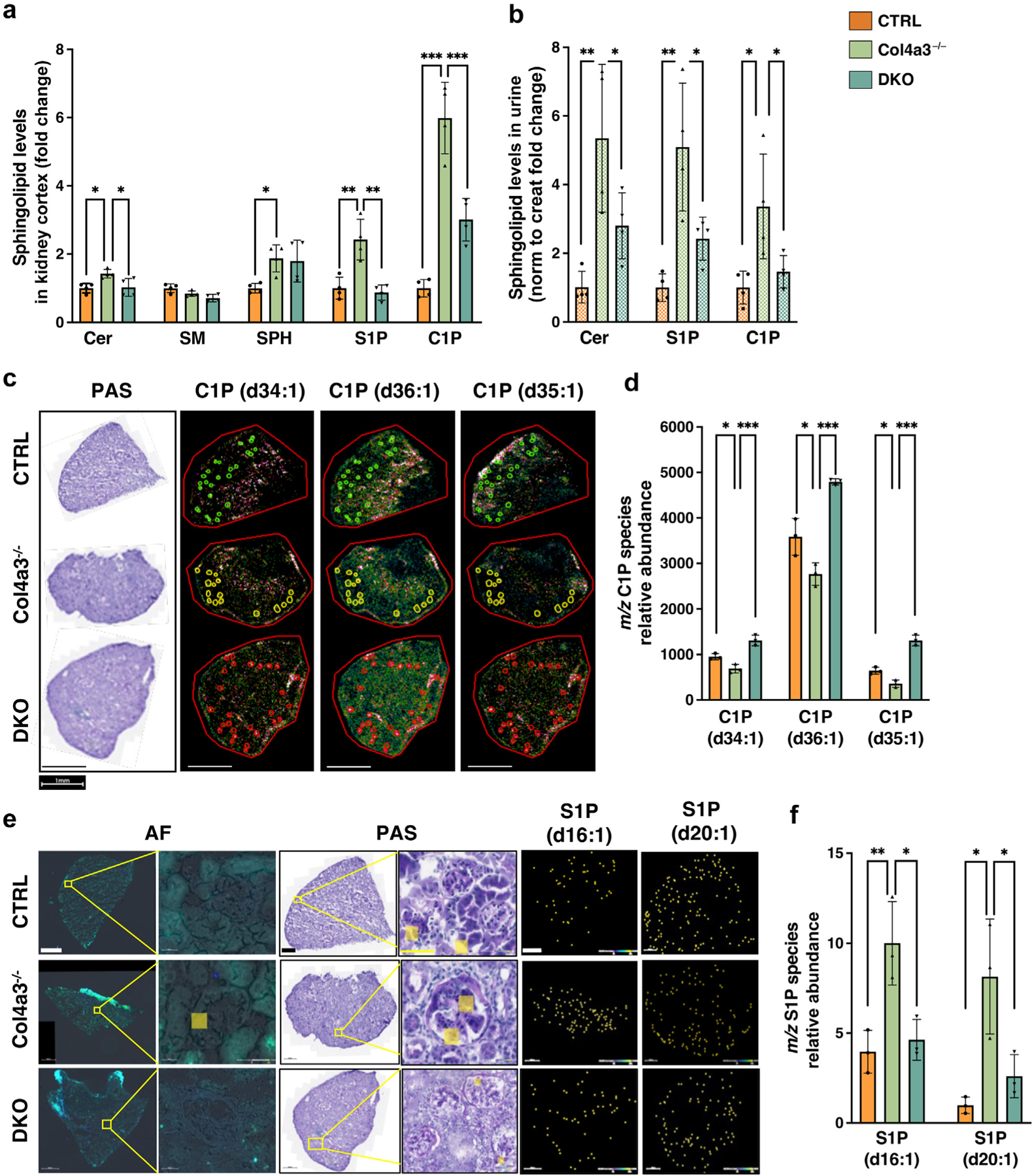
SMPDL3B expression affects the renal content of bioactive sphingolipids in Col4a3^−/−^ mice and Col4a3^−/−^ mice with podocyte-specific *Smpdl3b* deficiency. Four experimental groups of mice were used in the study: (i) wild-type control mice (CTRL, n = 4); (ii) mice with podocyte-specific *Smpdl3b* deletion (Smp^−/−^, n = 4); (iii) Col4a3^−/−^ mice (n = 4); and (iv) Col4a3^−/−^ mice with podocyte-specific *Smpdl3b* deletion (DKO, n = 4). (**a**) Liquid chromatography–mass spectrometry (LC-MS) analysis of total ceramide (Cer), total sphingomyelin (SM), sphingosine (SPH_, sphingosine-1-phosphate (S1P), and ceramide-1-phosphate (C1P) in kidney cortex isolated from mice. (**b**) LC-MS analysis of total Cer, S1P, and C1P in urine samples collected from all 4 groups of mice. (**c,d**) Spatial metabolomic of C1P species in glomeruli of CTRL, Col4a3^−/−^, and DKO mice: periodic acid–Schiff (PAS) and autofluorescence (AF) (the same tissue section) images (**c**) and bar graph analysis (**d**) of the most significantly changed C1P species. Each dot represents an independent glomerulus. Original magnification ×40; bar = 1 mm. Spatial metabolomic of S1P species in glomeruli of CTRL, Col4a3^−/−^, and DKO mice: PAS and overlayed AF (the same tissue section) images (**e**) and graph analysis (**f**) of the most significantly changed S1P species. Data are mean ± SD. *P < 0.05; **P < 0.01; ***P < 0.001. *P* values were calculated using 1-way analysis of variance (**a,b,d,f**). *m/z*, mass–charge ratio. To optimize viewing of this image, please see the online version of this article at www.kidney-international.org.

We next investigated whether alterations in renal sphingolipid levels may promote the early onset of proteinuria in AS mice. We performed lipidomic analysis of the bioactive sphingolipids in isolated kidney cortices and urine from 5-month-old CTRL, Col4a3^−/−^, and DKO mice by liquid chromatography–mass spectrometry. While no changes were found in the levels of total sphingomyelin, Col4a3^−/−^ mice demonstrated significant increase in total levels of ceramide, sphingosine, S1P, and, surprisingly, C1P, which were significantly decreased in mice with podocyte-specific *Smpdl3b* deletion (Figure 4a). Notably, we found C14:0, C16:0, C18:0, C18:1, C20:0, C20:1, C22:0, C22:1, C24:0, C24:1, and C26:1 C1P species to be significantly increased in Col4a3^−/−^ mice, while C14:0, C16:0, C20:1, C22:0, C22:1, C24:0, C24:1, C26:0, and C26:1 sphingomyelin species were significantly decreased in DKO mice compared to CTRL mice (Supplementary Data 4). Similarly, levels of total urinary ceramide, S1P, and C1P were significantly higher in Col4a3^−/−^ mice compared to CTRL mice (Figure 4b), where only ceramide species C16:0 and C22:0 were found significantly increased (Supplementary Data 5). Podocyte-specific deletion of Smpdl3b resulted in decreased total urinary ceramide, S1P, and C1P levels in DKO mice (Figure 4b), similar to data obtained in the kidney cortices from the same mice.

To further investigate the phenomenon of the observed increase in C1P levels, we performed spatial metabolomic analysis using matrix-assisted laser desorption ionization–mass spectrometry imaging specifically in glomeruli from CTRL, Col4a3^−/−^, and DKO mice. Indeed, levels of ceramide phosphate species (d34:1; d35:1; d36:1) were lower in glomeruli from Col4a3^−/−^ mice (Figure 4c). Quantitative analysis demonstrated that podocyte-specific *Smpdl3b* deficiency restored C1P levels in DKO mice compared to Col4a3^−/−^ mice (Figure 4d). While this is not the main focus of the present article, it is important to note that among the top 25 annotations with highest Variable Importance in Projection scores sphingomyelin, glucosylceramide, galactosylceramide, and gangliosides 3 and 4 appear (Supplementary Figure S7A). Many species of glucosylceramide and galactosylceramide are significantly regulated in Col4a3^−/−^ mice compared to CTRL mice, which may point to another important aspect of SMPDL3B function in glomeruli. Further metabolomic analysis also revealed clearly separated clusters of S1P species in glomeruli among CTRL, Col4a3^−/−^, and DKO mice (Supplementary Figure S7B). Spatial lipidomic analysis and overlayed autofluorescence (the same tissue section) or periodic acid–Schiff image (from a serial sections) of S1P (d16:1) and S1P (d20:1) were used as an example to confirm S1P accumulation in glomeruli of Col4a3^−/−^ mice, while levels are normal in DKO mice (Figure 4e and f), while heatmap analysis showed a significant upregulation of S1P species (d18:1, d17:1, d20:1, d19:1, d16:1) in Col4a3^−/−^ mice (Supplementary Figure S7C).

Taken together, these data suggest that SMPDL3B not only affects C1P availability but also regulates S1P levels, thereby contributing to the development of albuminuria in glomerular disease of nonmetabolic origin. Intriguingly, SMPDL3B overexpression seems to perturb glucosylceramide and galactosylceramide levels in AS mice, which opens a door to new avenues for investigating SMPDL3B function in podocytes. However, whether SMPDL3B overexpression is sufficient to cause glomerular disease progression remains elusive. To answer this question, we next generated mice with doxycycline-inducible podocyte-specific *Smpdl3b* overexpression.

### Doxycycline-inducible podocyte-specific *Smpdl3b* overexpression in mice does not cause proteinuria

**Figure 5 |:**
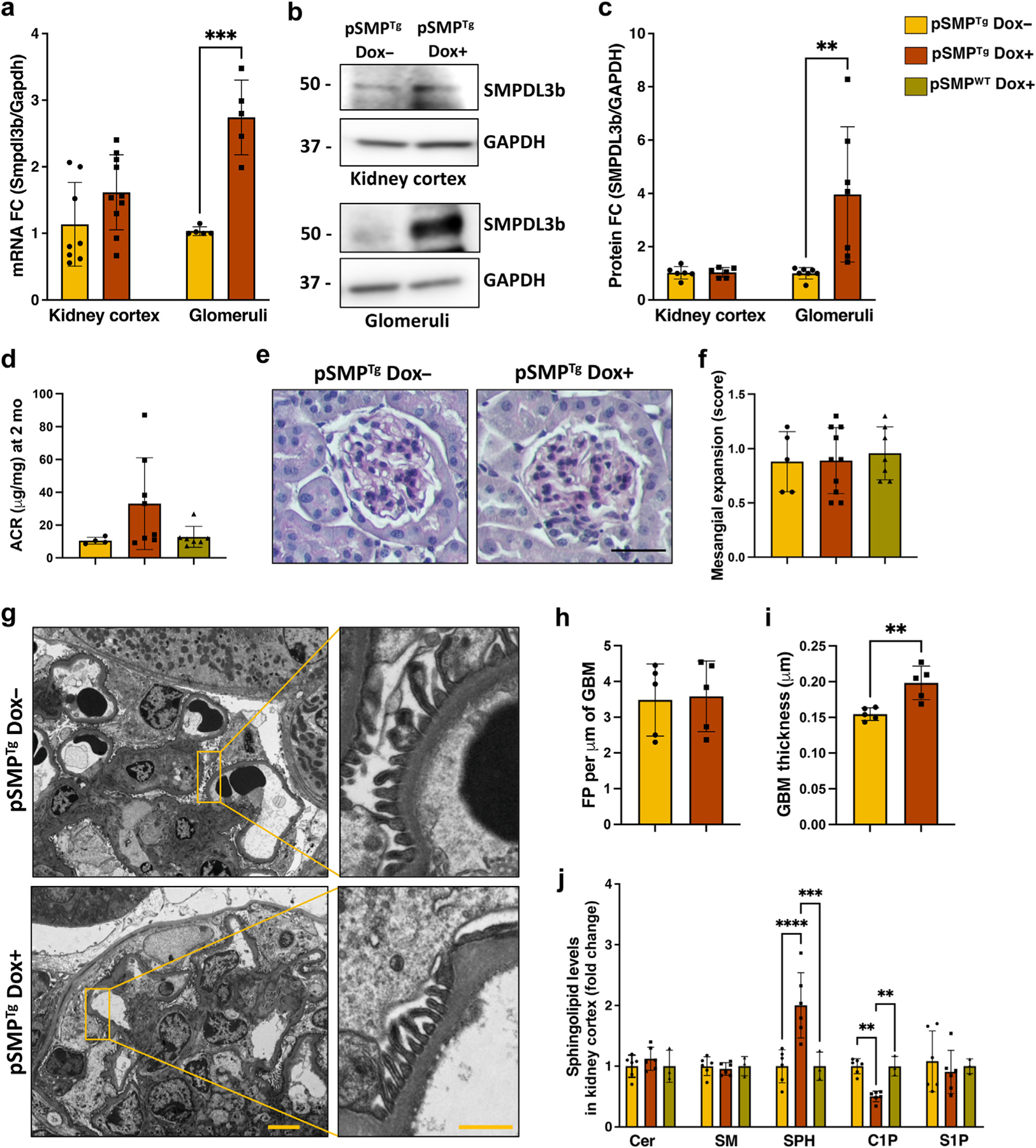
Mice with podocyte-specific *Smpdl3b* overexpression are phenotypically normal but have dysregulated sphingolipid metabolism and increased glomerular basement membrane (GBM) thickness. Three experimental groups of mice were used in the study: (i) uninducible mice with podocyte-specific *Smpdl3b* overexpression (pSMP^Tg^ Dox—,n = 5); (ii) doxycycline-inducible mice with podocyte-specific *Smpdl3b* overexpression (pSMP^Tg^ Dox+,n = 7); and (iii) doxycycline-inducible mice with intact Smpdl3b expression in podocytes (pSMP^WT^ Dox+, n = 7). (**a**) *Smpdl3b* mRNA expression levels in kidney cortex and glomeruli isolated from pSMP^Tg^ Dox— and pSMP^Tg^ Dox+ mice. Representative Western blot (**b**) and bar graph analysis (**c**) of SMPDL3B expression in kidney cortex and glomeruli isolated from pSMP^Tg^ Dox— and pSMP^Tg^ Dox+ mice. Pooled glomeruli isolated from 3–5 mice per pool used for each group. (**d**) Albumin–creatinine ratio (ACR) in all 3 groups of mice. Representative periodic acid–Schiff (PAS) staining () of 4-μm kidney sections (**e**) and bar graph analysis (**f**) in all 3 groups of mice. (**e**) Original magnification ×20; bar = 30 μm. (**g–i**) Representative semi-thin and scaled transmission electron microscopy images (**g**) and bar graph analysis of foot process (FP) effacement (**h**) and GBM thickness (**i**) in pSMP^Tg^ Dox— and pSMP^Tg^ Dox+ mice. (**g**) Original magnification ×10,000; bar = 4 μm and 1 μm for semi-thin and scaled images, respectively. (**j**) Liquid chromatography–mass spectrometry analysis of total ceramide (Cer), sphingomyelin (SM), sphingosine (SPH), ceramide-1-phosphate (C1P), and sphingosine-1-phosphate (S1P) in kidney cortex isolated from all 3 groups of mice. Data are mean ± SD. **P < 0.01; ***P < 0.001; ****P < 0.0001. *P* values were calculated using 2-tailed Student’s *t* test (**a,c,h,i**) and 1-way analysis of variance (**d,f,j**). To optimize viewing of this image, please see the online version of this article at www.kidney-international.org.

The generation of pSMP^Tg^ mice is described in detail in the Supplementary Extended Methods and is summarized in Supplementary Figure S2. SMPDL3B expression was induced in pSMP^Tg^ mice by feeding Dox+ chow for 30 days, and 8-week-old mice were analyzed. As expected, SMPDL3B expression mRNA (Figure 5a) and protein (Figure 5b and c) levels were significantly increased in glomeruli of pSMP^Tg^ mice with no changes in kidney cortex. However, this was not associated with changes in ACR (Figure 5d), body weight (Supplementary Figure S2C), or mesangial expansion score (Figure 5e and f). Interestingly, prolonged *Smpdl3b* expression for 16 months did not result in the development of proteinuria (Supplementary Figure S2D and E) or foot processes effacement (Figure 5g and h) in pSMP^Tg^ mice. However, unexpectedly, pSMP^Tg^ Dox+ mice were characterized by GBM thickening when compared to their controls (Figure 5g and i). Furthermore, lipidomic analysis demonstrated significantly increased levels of total sphingosine and significantly decreased levels of total C1P in kidney cortex of pSMP^Tg^ Dox+ mice compared to pSMP^Tg^ Dox- and pSMP^WT^ Dox+ controls with no changes in total ceramide, sphingomyelin, or S1P (Figure 5j). While most of the analyzed C1P species were downregulated in pSMP^Tg^ Dox+ mice, C18:0 and C18:1 species were the mostly affected (Supplementary Data 6). Illumina RNA-sequencing (RNA-seq) analysis also showed disturbance in many genes regulating sphingolipid metabolism in human podocytes with SMPDL3B overexpression (Supplementary Figure S2F). Because the only abnormality observed in pSMP^Tg^ Dox+ mice was an increase in GBM thickness, among all the renal parameters assessed (Figure 5g and i), while DKO mice exhibit a significant decrease in GBM thickness compared to Col4a3−/− mice (Figure 3h), we next explored the role of SMPDL3B in regulating GBM thickness.

### SMPDL3B overexpression causes GBM and podocyte softening

**Figure 6 |:**
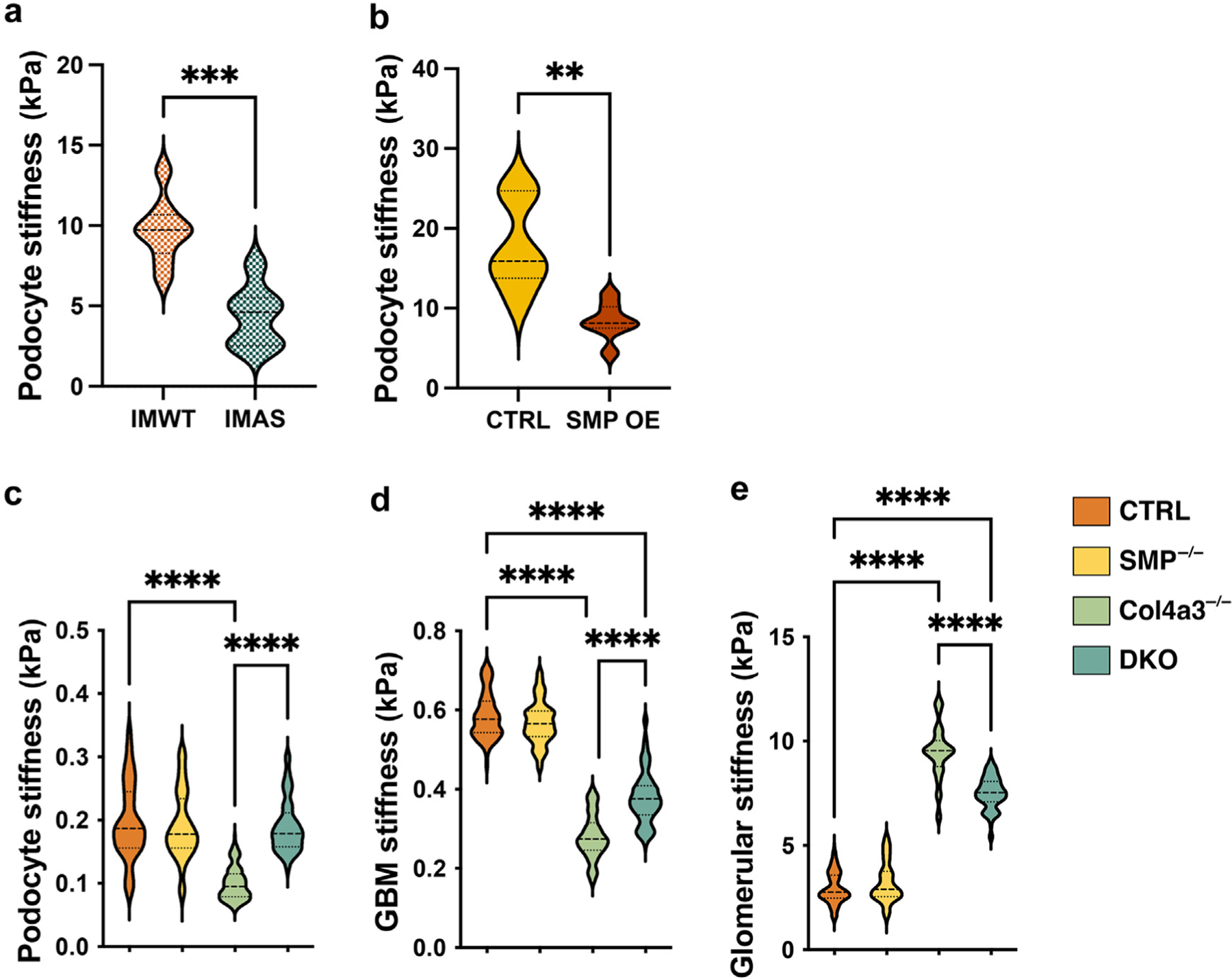
SMDP3LB overexpression affects biomechanical properties of podocytes and glomerular basement membrane (GBM) *in vitro* and *in vivo*. (**a**) Violin plot of mechanical properties (stiffness) measurement as Young’s module (YM, kPa) in immortalized murine podocytes isolated from Col4a3^+/+^ (immortalized murine podocytes isolated from wild-type [IMWT]) and Col4a3^−/−^ (immortalized murine podocytes isolated from Col4a3^−/−^ [IMAS]) mice. (**b**) Violin plot of mechanical properties (stiffness) measurement as YM (kPa) in immortalized human podocytes with normal (CTRL) or increased (SMP overexpressed [OE]) SMPDL3B expression. Violin plots of mechanical properties (stiffness) measurement as YM (kPa) in podocytes (**c**), GBM (**d**), and glomeruli (**e**) from (i) wild-type control mice (CTRL, n = 5), (ii) mice with podocyte-specific *Smpdl3b* deletion (Smp^−/−^, n = 5); (iii) Col4a3^−/−^ mice (n = 5); and (iv) Col4a3^−/−^ mice with podocyte-specific *Smpdl3b* deletion (DKO, n = 5). Error bars indicate 95% CI. **P < 0.01; ***P < 0.001; ****P < 0.0001. *P* values were calculated using 2-tailed Student’s *t* test (**a,b**) and 1-way analysis of variance (**c–e**).

We performed a series of atomic force microscopy experiments exploring podocytes and GBM stiffness using our *in vivo* and *in vitro* models. Both, murine IMAS and human podocytes with SMPDL3B overexpression demonstrated a significant decrease in cell stiffness compared to their controls (Figure 6a and b). Illumina RNA-seq analysis highlighted downregulation of many genes involved in regulating GBM integrity, including collagens and laminins (Supplementary Figure S2G). Importantly, atomic force microscopy analysis of kidneys from Col4a3^−/−^ mice with podocyte specific *Smpdl3b* KO (DKO) showed increased podocyte (Figure 6c) and GBM stiffness (Figure 6d) compared to Col4a3^−/−^ mice. However, overall glomerular stiffness was increased in Col4a3^−/−^ mice while DKO mice have significantly decreased glomerular stiffness compared to Col4a3^−/−^ mice (Figure 6e). These findings indicate that SMPDL3B plays a crucial role in regulating glomerular biomechanics, which may partially explain why foot processes remain preserved in DKO mice and why these mice do not develop proteinuria. Moreover, in Col4a3^−/−^ mice, glomerular *Smpdl3b* overexpression is observed as early as at 4-week-old mice, suggesting that role of SMPDL3B overexpression in younger mice requires further investigation. To further investigate the role of S1P/C1P/SMPDL3B axis in podocytes, we conducted a series of experiments involving both *in vitro* and *in vivo* treatments with C1P or S1P.

### S1P but not C1P treatment of human podocytes causes apoptosis *in vitro* and S1P treatment worsens proteinuria and apoptosis *in vivo*

**Figure 7 |:**
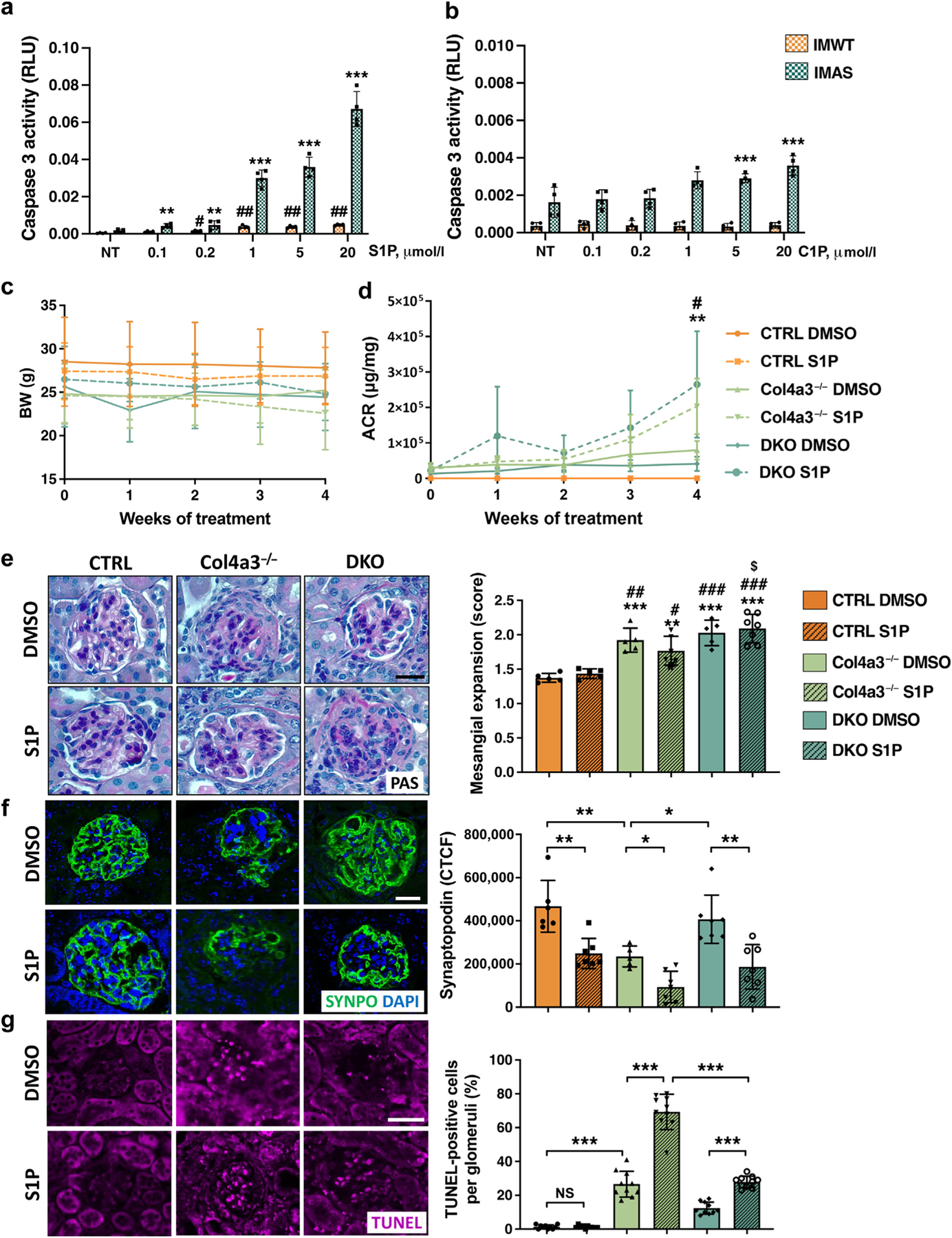
Exogenous sphingosine-1-phosphate (S1P) increases apoptosis both *in vitro* and *in vivo*, accompanied by worsened proteinuria and decreased synaptopodin expression, particularly when SMPDL3B is overexpressed. *In vitro*, immortalized murine podocytes isolated from wild-type (IMWT) and from Col4a3^−/−^ mice (IMAS) were treated with increased doses of S1P or ceramide-1-phosphate (C1P) (n = 4 per group). (**a**) Caspase 3 activity in podocytes treated with vehicle (albumin only; nontreated [NT]) or increased albumin-bound S1P doses. **P < 0.01, ***P < 0.001, 1-tailed Student’s *t* test, when S1P-treated IMAS compared to NT IMAS. ^#^*P* < 0.05, ^##^*P* < 0.01, 2-tailed Student’s *t* test, when S1P-treated compared to NT IMWT. (**b**) Caspase 3 activity in podocytes treated with vehicle (dimethyl sulfoxide [DMSO] only; NT) or increased C1P doses. ***P < 0.001, 1-tailed Student’s *t* test, when C1P-treated IMAS compared to NT IMAS. *In vivo*, 6 groups of mice were used: (i) control (CTRL) mice treated with 5% DMSO in 4 mg/ml albumin (CTRL DMSO, n = 5); (ii) control mice treated with 0.04 mg/kg S1P in 4 mg/ml albumin (CTRL S1P, n = 7); (iii) Col4a3^−/−^ treated with 5% DMSO in 4 mg/ml albumin (Col4a3^−/−^ DMSO, n = 5); (iv) Col4a3^−/−^ mice treated with 0.04 mg/kg S1P in 4 mg/ml albumin (Col4a3^−/−^ S1P, n = 7); (v) Col4a3^−/−^ mice with podocyte-specific *Smpdl3b* deletion (DKO) treated with 5% DMSO in 4 mg/ml albumin (DKO DMSO, n = 5); (vi) DKO mice treated with 0.0002 mg/kg S1P in 4 mg/ml albumin (DKO S1P, n = 7). Body weight (**c**) and albumin–creatinine ratio (ACR) (**d**) in all 6 groups of mice at all time points. **P < 0.01 when Col4a3^−/−^ DMSO compared to Col4a3^−/−^ S1P; ^#^*P* < 0.05 when DKO DMSO compared to DKO S1P; 2-way analysis of variance (ANOVA). (**e**) Representative periodic acid–Schiff (PAS) staining of paraffin-embedded kidney sections (4 μm) and bar graph analysis of the scores for mesangial expansion in all 6 groups of mice. Original magnification ×40; bar = 30 μm. *P < 0.01, ***P < 0.001 when compared to CTRL DMSO; ^#^*P* < 0.05, ^##^*P* < 0.01, ^###^*P* < 0.001 when compared to CTRL S1P; ^$^*P* < 0.05 when compared to Col4a3^−/−^ S1P; 1-way ANOVA. (**f**) Representative staining for synaptopodin (SYNPO) (*green*) and 4ʹ,6-diamidino-2-phenylindole (DAPI) (blue) of paraffin-embedded kidney sections (4 μm) and bar graph analysis of synaptopodin corrected total cell fluorescence (CTCF) in all 6 groups of mice. Original magnification ×40; bar = 20 μm. (**g**) Representative terminal deoxynucleotidyl transferase–mediated dUTP nick end-labeling (TUNEL) BrdU-red staining in 4-μm paraffin-embedded kidney sections and bar graph analysis of TUNEL-positive cells in all 6 groups of mice. Original magnification ×20; bar = 20 μm. Data are mean ± SD. *P* values were calculated using 2-way ANOVA (**f,g**). NS, not significant; RLU, relative light units. To optimize viewing of this image, please see the online version of this article at www.kidney-international.org.

S1P is generally considered to have antiapoptotic effects and is involved in promoting cell survival and proliferation, while ceramide is known to have proapoptotic effects. However, some reports suggest that S1P can also trigger apoptosis.^20,40^ Interestingly, the physiological relevance of the prosurvival effect of C1P is underscored by the demonstration that intracellular levels of C1P are substantially decreased in apoptotic macrophages,^41^ similar to what we observed in podocytes isolated from Col4a3^−/−^ mice (Supplementary Figure S3F). Therefore, we next tested whether exogenous S1P or C1P supplementation causes apoptosis. Using immortalized murine podocytes isolated from wild type (control) (IMWT) mice and from Col4a3^−/−^ (IMAS) mice,^34^ we demonstrate that albumin-bound S1P treatment causes a dose-dependent increase in Caspase 3 activity in both IMWT and IMAS podocytes (Figure 7a). Interestingly, IMAS podocytes demonstrated Caspase 3 levels 4 to 13 times higher than those of IMWT podocytes, suggesting that increased SMPDL3B expression in IMAS podocytes is associated with a higher susceptibility to S1P-associated injury. However, treatment with increased doses of C1P resulted in significant increase in Caspase 3 levels in IMAS but not in IMWT podocytes (Figure 7b).

Based on our *in vitro* observations, we performed a series of *in vivo* experiments, where 16-week-old CTRL, Col4a3^−/−^, and DKO mice were treated by daily i.p. injection of albumin-bound S1P or DMSO for 4 weeks. To control for the quality of i.p. S1P injections, we used S1P (C17:0), the most stable species that can be used as an internal standard for the quantification of S1P (d18:1). Liquid chromatography–mass spectrometry analysis confirmed the quality of injections as higher levels of S1P (C17:0) were observed in kidney cortices of S1P-injected mice compared to DMSO-injected controls (Supplementary Figure S8). S1P treatment did not result in any significant changes in body weight between the groups of mice (Figure 7c). S1P treatment of Col4a3^−/−^ and DKO resulted in worsen ACR levels (Figure 7d) but did not increase mesangial expansion compared to untreated CTRL mice (Figure 7e). Moreover, Col4a3^−/−^ mice demonstrated reduced glomerular synaptopodin (a marker of podocyte actin cytoskeleton integrity) expression compared to CTRL mice, while synaptopodin levels were restored in DKO mice (Figure 7f). S1P treatment resulted in an even more severe reduction of synaptopodin expression in Col4a3^−/−^ mice and significantly reduced synaptopodin levels in DKO and CTRL mice (Figure 7f), suggesting S1P might mediate podocyte injury by affecting the podocyte actin cytoskeletal integrity. To assess the glomerular apoptosis levels *in vivo*, terminal deoxynucleotidyl transferase–mediated dUTP nick end-labeling assay was used. We found that the apoptosis levels are increased in Col4a3^−/−^ mice at the baseline, and that S1P treatment aggravated apoptosis levels in both Col4a3^−/−^ and DKO (Figure 7g) but not in CTRL mice. Importantly, S1P-treated DKO mice showed significantly lower apoptosis levels compared to S1P-treated Col4a3^−/−^ mice (Figure 7g), suggesting that another hit such as increased SMDPL3B levels is needed to cause podocyte injury *in vivo*.

## DISCUSSION

**Figure 8 |:**
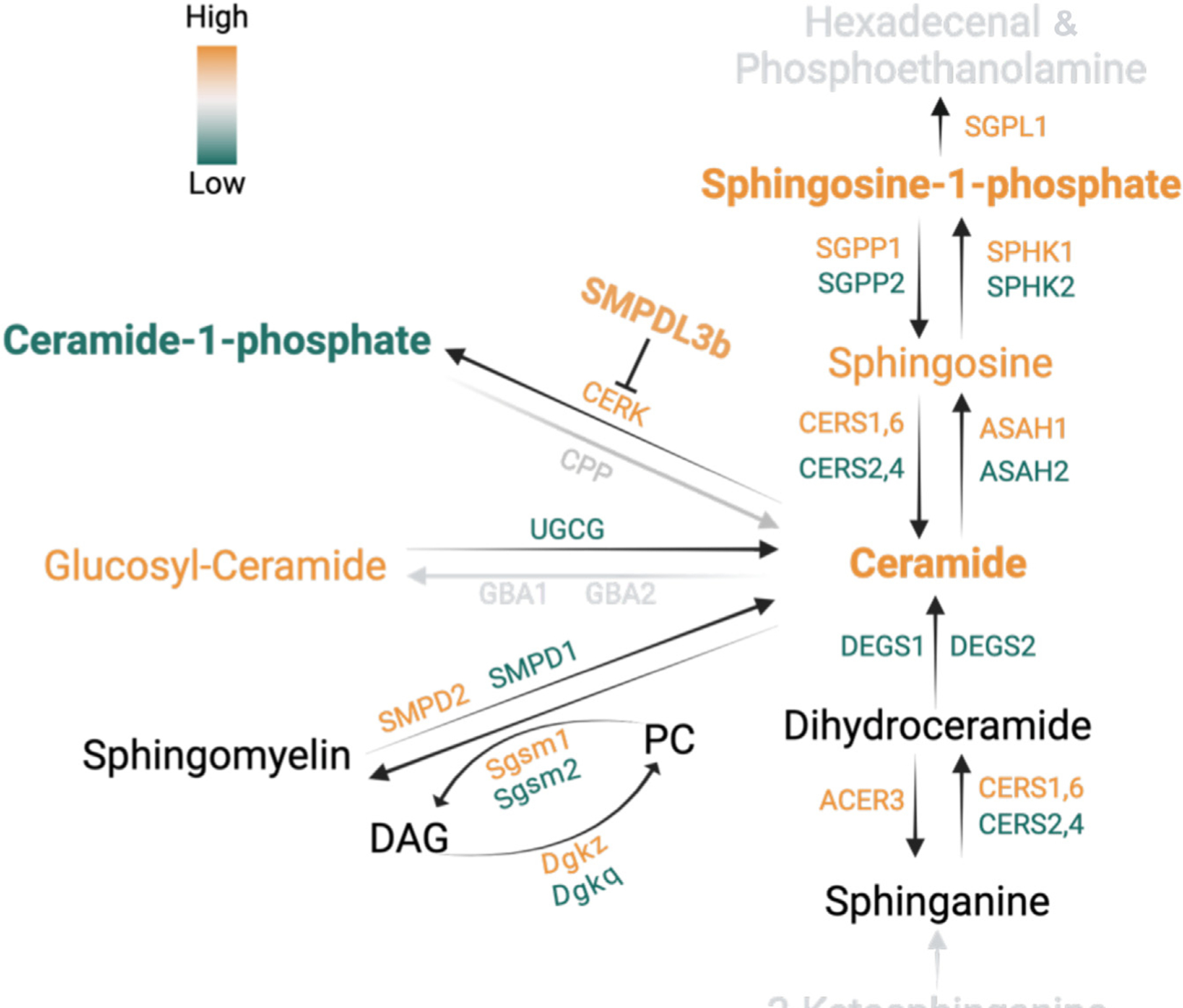
The summary of study findings. Increases or decreases in the levels and activity of the examined sphingolipids and related enzymes are highlighted in orange and green, respectively. Sphingolipids and related enzymes with no significant changes are shown in *black*. Sphingolipids and related enzymes that were not examined in this study are shown in *gray*. CoA, coenzyme A; DAG, diacylglycerol; PC, phosphatidylcholine. Created using BioRender.com. Mitrofanov, A. (2025) https://BioRender.com/i26g266.

Here, we demonstrate for the first time a sphingolipid-dependent decoupling between proteinuria and kidney disease progression in Col4a3^−/−^ mice, a model of experimental AS, which is summarized in Figure 8. Similar to what we reported in diabetic kidney disease (DKD), a metabolic disorder affecting the GBM,^28^ glomerular SMPDL3B expression levels are also increased in the mouse model of experimental AS, a nonmetabolic disorder affecting the GBM. Interestingly, while podocyte-specific *Smpdl3b* deficiency is sufficient to reduce both proteinuria and disease progression in experimental DKD,^18^ reduced proteinuria in the absence of renal function improvement was observed in Col4a3^−/−^ mice with podocyte-specific *Smpdl3b* deficiency. These findings challenge the role of proteinuria as a valid surrogate end point for clinical trials development in AS, as recently suggested with the use of sparsentan in focal and segmental glomerulosclerosis, where a reduction of proteinuria was not accompanied by a difference in estimated glomerular filtration rate slope.^42^

SMPDL3B is a glycophosphatidylinositol-anchored protein^43,44^ that has been shown to regulate innate immune responses and plasma membrane fluidity in macrophages^31^ and to contribute to the progression of DKD^18,28^ and focal segmental glomerulosclerosis.^29^ Our prior reports showed that SMPDL3B overexpression is associated with increased levels of S1P^37^ and decreased levels of C1P^18^ *in vitro.* In particular, SMPDL3B regulates C1P levels via interaction with ceramide kinase preventing conversion of ceramide into C1P.^30^ These data suggest that SMPDL3B may function as a switch that regulates the content of bioactive sphingolipids in the kidney. Using 2 different mouse models of experimental AS, we demonstrated for the first time that increased glomerular SMPDL3B expression *in vivo* is associated with increased glomerular levels of S1P.

S1P is an active metabolite involved in the regulation of many physiological processes and in the pathogenesis of many diseases, including autoimmune diseases, cancer, atherosclerosis, diabetes, and osteoporosis.^20^ The role of S1P in kidney disease progression has been extensively studied in the context of acute kidney injury,^45–48^ while its role in CKD remains elusive. Several recent reports showed that mutations in the gene encoding for S1P lyase (*SGPL1*) results in accumulation of S1P, severe disease progression, and is associated with nephrotic syndrome,^23,25,49,50^ similar to what was described in *Sgpl1*-deficient mice.^51^ The involvement of S1P in many cellular physiological processes and diseases may be a result of its action as both an intracellular and extracellular signaling molecule, which was recently reviewed.^21^ In support of this idea, our data showed increased S1P levels, not only in kidney cortex and glomeruli, but in the urine of Col4a3^−/−^ mice.

While we did not describe the specific mechanisms by which SMPDL3B influences the S1P content, we observed that several enzymes involved in S1P synthesis (SPHK1 and SPHK2) or degradation (reversibly by SPP1 and SPP2 or irreversibly by SGPL1) were differentially regulated in Col4a3^−/−^ mice, suggesting that SMPDL3B may modulate S1P levels through its effects on the expression or activity of these enzymes. While these data point at intracellular S1P as a potential mediator of injury in our model, exogenous administration of S1P significantly increased albuminuria in Col4a3^−/−^ but not in control mice, suggesting the need for a second hit or the existence of compensatory mechanisms *in vivo*. However, the exact mechanism by which S1P increases albuminuria in Col4a3^−/−^ but not in control mice remains to be established. *In vitro*, treatment of murine podocyte with gradually increasing concentrations of S1P resulted in increased Caspase 3 activity in both wild-type and Col4a3^−/−^ podocytes. While these data may seem to argue against a common paradigm that S1P stimulates cell growth and survival in cancer cells, this seems to be cell specific because S1P was also shown to trigger apoptosis in human hepatic myofibroblasts^40^ or B16 mouse melanoma cells.^52^ Interestingly, studies in human cardiac fibroblasts also demonstrated that extracellular S1P can induce apoptosis via stimulation of S1PR1/S1PR3.^53^ Taken together, these studies suggest that extracellular and intracellular S1P may have different roles in promoting cell survival or apoptosis depending on the cell type. Furthermore, S1P receptor selectivity may offer another potential explanation for these differential effects. For example, it has been demonstrated that under physiological conditions, S1P preferentially binds to S1PR1, while S1P preferentially binds to S1PR2 and induces F-actin disorganization when S1P is available in excess.^54^ Our own data showing that exogenous S1P affects synaptopodin expression and cytoskeletal proteins organization in glomeruli of Col4a3^−/−^ and DKO mice, lead us to speculate that S1P may contribute to the development of proteinuria and foot processes effacement. However, how S1PR signaling is altered in Col4a3^−/−^ mice requires further investigation. Besides S1P, spatial lipidomic analysis also revealed significantly decreased C1P species in glomeruli from Col4a3^−/−^ mice, suggesting that bioactive sphingolipids may represent potential new therapeutic agents and or therapeutic targets for the treatment of patients with AS and more broadly with CKD. In fact, sphingolipid metabolism has been recognized as a therapeutic option to treat Alzheimer’s disease,^55^ some types of cancer (reviewed in Ogretmen^56^), and diabetes^57^; therefore, testing compounds targeting the SMPDL3B-S1P axis that could reduce podocyte injury would be beneficial.

The novel finding that SMPDL3B may contribute to GBM and podocyte mechanical properties by influencing matrix composition is important because it may lead to the discovery of new therapeutic strategies for the treatment of patients with AS. Indeed, cellular sense of mechanical cues has an important role in regulating many pathways, including proliferation and apoptosis. Sphingolipids are closely linked to cell stiffness, because their composition and metabolism within the cell membrane can significantly influence its physical properties, with higher levels of certain sphingolipids, particularly ceramides (reviewed in Ya’ar Bar *et al.*^58^), generally leading to increased cell stiffness due to their ability to pack tightly and create more rigid membrane domains.^59^ In contrast, S1P is generally associated with decreased cell stiffness due to its involvement in cell migration and proliferation pathways (reviewed in Iqbal *et al.*^60^), which is in alignment with the finding of decreased stiffness in podocytes from mice with AS. One possibility that these changes in GBM stiffness in mice with experimental AS contribute to a metabolic shift in podocytes from glycolysis to fatty acids oxidation and reduced stress fibers as recently described by us.^61^

Mechanistically, we propose that excess SMPDL3B alters the distribution of S1P transporters (such as Mfsd2b and Spns2) at the plasma membrane, leading to S1P accumulation in podocytes and potentially tubular cells, thereby contributing to kidney dysfunction. Deleting Smpdl3b in podocytes improves membrane biomechanics and reduces intracellular S1P levels, resulting in better podocyte integrity and reduced proteinuria. However, SMPDL3B expression remains elevated in tubular cells, which may disrupt S1P signaling and explain the lack of improvement in overall renal function. We further speculate that deleting SMPDL3B in both podocytes and tubular cells could lead to improvements in both proteinuria and renal function. However, further studies are needed to prove this hypothesis. Additionally, we and others have demonstrated that in diabetic nephropathy^62^ and primary glomerular diseases (focal segmental glomerular sclerosis, minimal change disease, membranous nephropathy, or immunoglobulin A nephropathy),^63^ the expression of apolipoprotein M, the physiological carrier of S1P, is dramatically reduced. This supports the notion that S1P-mediated signaling is actively involved in glomerular disease progression.

It is important to note that our study demonstrated decreased expression of glucosylceramides and 3’-sulfo galactosylceramides, the most abundant glycosphingolipids, in glomeruli of mice with AS. Being synthesized from ceramide by glucosylceramide synthase (Ugcg) into glucosylceramides or by galactosylceramide synthase (Ugt8a) into galactosylceramides, these glycosphingolipids are important cell type–specific components of lipid raft domains and thus they may contribute to podocyte injury in AS. Interestingly, in patients with Fabry’s disease, the partial or complete loss of α-galactosidase A activity results in accumulation of globotriaosylceramide throughout the kidney, including podocytes,^64^ thereby contributing to the renal phenotype observed in these patients. In DKD, glycosphingolipids were shown to cause insulin resistance, whereby the ganglioside GM3 interacts with insulin receptor causing its sequestration from cholesterol-enriched rafts^65,66^ and insulin signaling deficiency. While the role of SMPDL3B in regulation of glycosphingolipids expression remains unexplored, our data suggest that podocyte-specific deletion of SMDPL3B in AS mice is associated with normalized levels of glucosylceramides and 3ʹ-sulfo galactosylceramides. Therefore, the contribution of SMPDL3B to glycosphingolipids metabolism warrants further investigation.

In summary, our study identifies that targeting SMPDL3B may improve glomerular function and reduce proteinuria in AS but may not be sufficient to halt CKD progression, which underscores the need for combined therapeutic strategies addressing both glomerular and tubulointerstitial compartments. Limitations of this study include the complexity of the pathway studied, the potential cross-talk between glomerular cells and the lack of deep analysis of tubulointerstitial compartment. Thus, while podocyte-specific SMPDL3B deficiency is sufficient to rescue from proteinuria but does not halt CKD progression, the role of SMPDL3B derived from other glomerular cells and tubular cells in mediating renal fibrosis was not investigated. Additional studies with tubular-specific SMPDL3B KO mice may need to be performed. In addition, while we demonstrate that SMPDL3B expression affects S1P and C1P levels, we did not demonstrate that S1P is the sole downstream mediator of SMPDL3B function. Although this is suggested by the fact that while DKO mice are protected from proteinuria, exogenous S1P administration brings proteinuria in DKD mice at the same high level as in Col4a3^−/−^ mice. Another major limitation is the lack of dedicated pharmacokinetics studies on S1P tissue distribution and half-life. Therefore, further studies to investigate the precise mechanisms leading to sphingolipid imbalances in kidney diseases and to test the clinical efficacy and superiority to the standard of care of agents modulating renal bioactive sphingolipids are warranted.

## Supplementary Material

Supplemental Materials

Supplementary Data 1

Supplementary Data 2

Supplementary Data 3

Supplementary Data 4

Supplementary Data 5

Supplementary Data 6

Supplementary material is available online at www.kidney-international.org.

### DATA STATEMENT

The data on bulk RNA-seq analysis from immortalized murine podocytes isolated from Col4a3^−/−^ mice supporting the findings of this study are openly available in the National Center for Biotechnology Information’s Gene Expression Omnibus repository (GSE274298). Additional data on the whole-transcript array from kidneys isolated from 8-week-old Col4a3^−/−^ mice, whole-transcript array data on urine-derived podocytes from 3 patients with AS, and digital spatial profiling to describe glomerular SMPDL3B expression in kidney biopsies from patients with AS were derived from the following resources available in the public domain: GSE109777 (https://www.ncbi.nlm.nih.gov/geo/query/acc.cgi?acc=GSE109777), GSE134011 (https://www.ncbi.nlm.nih.gov/geo/query/acc.cgi?acc=GSE134011), and GSE255265 (https://www.ncbi.nlm.nih.gov/geo/query/acc.cgi?acc=GSE255265), respectively, from the National Center for Biotechnology Information’s Gene Expression Omnibus repository. We used the ARRIVE1 checklist when writing our report.^67^

## Translational Statement

The role of sphingolipids in the pathogenesis of proteinuria and renal failure in Alport syndrome (AS) remains elusive. This study reveals that SMPDL3B overexpression in experimental AS (Col4a3 knockout mice) disrupts sphingolipid metabolism and compromises glomerular basement membrane and podocyte mechanical integrity, contributing to proteinuria. Podocyte-specific *Smpld3b* deletion in Col4a3 knockout mice ameliorates glomerular structure and reduces proteinuria yet does not prevent progression to renal failure. These findings suggest that while targeting SMPDL3B may improve glomerular function and reduce proteinuria in AS, this intervention may not be sufficient to halt chronic kidney disease progression. This dissociation highlights the complexity of AS pathogenesis and underscores the need for combined therapeutic strategies addressing both glomerular and tubulointerstitial compartments.
