# Supplementary material for: The enzyme SMPDL3b in podocytes decouples proteinuria from chronic kidney disease progression in experimental Alport Syndrome
Source: Kidney Int. Author manuscript; Available in PMC 2026 May 30. (PMC12724640; doi:10.1016/j.kint.2025.04.024)
Supplement: Supplemental Materials [file NIHMS2120004-supplement-Supplemental_Materials.pdf]

SUPPLEMENTAL MATERIALS

SMPDL3b in Podocytes: Decoupling Proteinuria from CKD Progression in Experimental Alport Syndrome

Alla Mitrofanova, Antonio M. Fontanella, Judith Molina, Guanshi Zhang, Shamroop K. Mallela, Luisa Ulloa Severino, Javier Varona Santos J., Matthew Tolerico, Rachel Njeim, Wadih Issa, Maria Boulina, Arianna Carrazco, Veronika Semenova, Yiqin Zuo, Maria Ficarella, Jin Ju Kim, Alexis Sloan, Kumar Sharma, Darren Yuen, Laura Perin, George W. Burke, Alessia Fornoni and Sandra Merscher

Table of Contents

SUPPLEMENTARY EXTENDED METHODS....... 2
SUPPLEMENTARY TABLES 23
SUPPLEMENTARY DATA 24
SUPPLEMENTARY REFERENCES 25

SUPPLEMENTARY EXTENDED METHODS

Reagents and materials

RPMI 1640 media for podocytes culture (#10-040-CV), insulin-transferrin-selenium (ITS, #25-800-CR) and collagen type I (#354236) were obtained from Corning (Corning, NY, USA). Fetal bovine serum (#26140-079), penicillin/streptomycin (#15140-163), and DMEM/F12 (1:1) media for murine podocytes culture (#11320-033) were purchased from Gibco | ThermoFisher Scientific (Emeryville, CA, USA). Myc-DDK-SMPDL3b cDNA vector (#RC217688L3V) was obtained from Origene (Rockville, MD). Ase I (#R0526S) enzyme was purchased from New England Biolabs (Ipswich, MA). RNAeasy kit (#74106), DNAeasy kit (#69506), Gel Extraction kit (#28704) and PCR purification kit (#28106) were obtained from Qiagen (Germantown, MD, USA). GoTaq green master mix (#M7123) was purchased from Promega (Madison, WI, USA). qScript cDNA Supermix (#95048-500) and SYBR green ROX mix (95073-012) were obtained from QuantaBio (Beverley, MA, USA). Pierce^{TM} BCA Protein Assay kit (#23225) and ProLong™ Gold Antifade Mountant with DAPI (#P36935) were purchased from Invitrogen | ThermoFisher Scientific (Emeryville, CA, USA). SDS-PAGE gels (#456-1094) and Trans-Blot Turbo Transfer Membranes (#1704156) for Western blot were purchased from Bio-Rad (Hercules, CA, USA). Albumin ELISA kit (#E90-134) was obtained from Bethyl Lab (Montgomery, TX, USA) and creatinine kit (#0420-500) was obtained from Stanbio (Boerne, TX, USA). Sieve strainers (#352350 – 70 μm; #352360 – 100 μm) were purchased from Falcon Biological (Kernville, CA, USA). TUNEL assay kit (#ab66110) were obtained from Abcam (Branford, CT, USA). Sphingosin-1-phosphate (d17:1, #860641) and ceramide-1-phosphate (d18:1/16:0, #860533) were ordered at Avanti Polar Lipids (Birmingham, AL, USA). 1,5-Dihydroxybenzoic acid (DAN≥99%, #149357), TBS (#T5912), Mayer's hematoxylin (MHS32), ethanol (#E7030) and xylene (#185566) were ordered from Millipore Sigma (Burlington, MA, USA). Indium tin oxide (ITO) glass slides (#MFPRM7462243) were ordered from Bruker Daltonics (Bremen, Germany). Hydrogen peroxidase blocking reagent (#GR3415498), DAB substrate kit (#GR3422585) and TUNEL assay kit (#ab66110) were obtained from Abcam (Cambridge, MA).

Cell culture

Immortalized murine podocytes isolated from control mice (IMWT) and mice with experimental Alport syndrome (IMAS) were generated as previously described[1], and were cultured on type I collagen-coated flasks in DMEM/F12 media supplemented with 20% FBS, 1% penicillin/streptomycin and 1% interferon-γ at 33°C to promote cells proliferation. Cells were grown to confluence 70-80%, trypsinized and reseeded in fresh flasks at a dilution 200,000 cells per 10 mL of DMEM/F12 media without interferon-γ, followed by thermoshifting to 37°C to promote cell differentiation.

A human podocyte cell lines transfected with a thermosensitive SV40-T construct were cultured on type I collagen-coated flasks in RPMI media supplemented with 10% FBS, 1% penicillin/streptomycin,

and 1% ITS at 33 °C to promote cells proliferation[2]. Before thermoswitching to 37 °C to promote cells differentiation, cells were grown to confluence 70–80% and trypsinized and reseeded in fresh flasks at a dilution 200,000 cells per 10 ml of RPMI media without ITS. The origin of normal human podocytes (control) is a kind gift of Dr. Jochen Reiser, Rush University, Chicago. SMPDL3b overexpression human podocytes were developed by Dr. Fornoni and validated previously[3].

Podocytes treatment

Immortalized murine podocytes were differentiated for 14 days and serum starved for 24h starting day 13. Treatment with sphingosine-1-phosphate (S1P d17:1) was performed at concentrations 0.1, 0.2, 1, 5 and 20 μM for 2 h at 37°C in FBS free DMEM/F12 media. S1P stock solution was solubilized in heated DMSO solution (2.6 mM) followed by dilution in 4% fatty acids free albumin solution to prepare the final solution for the treatment. Treatment with ceramide-1-phoshate (S1P d16:0) 0.1, 0.2, 1, 5 and 20 μM for 1 h at 37°C in FBS free DMEM/F12 media. C1P stock solution (final concentration 1.47 mM) was prepared by sonication of C1P in sterile nanopure water on ice using a probe sonicator, until a clear dispersion was obtained[4]. Podocytes treated with vehicle served as a control.

RNA extraction

Mouse kidney tissues and glomeruli after isolation were snap-frozen in liquid nitrogen and stored at -80°C until processing. Pellets from human and murine podocytes were kept in RTL+β-mercaptoetanol buffer (Qiagen kit) at -80°C until processing. RNA was isolated using RNAeasy kit according to the manufacture's protocol.

PCR analysis

250 ng RNA was reverse transcribed using the qScript cDNA Supermix according to the manufacturer's protocol. qRT-PCR was performed using SYBR green ROX mix. Agarose gel PCR for genotyping was performed using GoTaq green master mix at 58 °C, 35 cycles. Primer sequences used in the study are listed in Supplementary Table 1.

Bulk RNA-seq

RNA was isolated from control (IMWT) and Alport Syndrome (IMAS) immortalized murine podocytes using RNeasy Mini kit. RNA was further processed for sequencing by the John P. Hussman Institute for Human Genomics Core Facility at University of Miami. mRNA-seq libraries were prepared using the TruSeq mRNA stranded LT (Illumina kit). The samples were sequenced using the NovaSeq 6000 system sequencing with 2x100 cycle (paired read), high output mode (expected a minimum of 30 million reads).

Raw sequence data were processed by the on-instrument Real Time Analysis software (v.2.7.7) to base call files. These were converted to de-multiplexed FASTQ files with the Illumina supplied scripts in

the BCL2FASTQ software (v2.17). The quality of the reads was determined with FASTQC software for per base sequence quality, duplication rates, and overrepresented k-mers. Illumina adapters were trimmed from the ends of the reads using Trim Galore! package. Reads were aligned to the human reference genome (hg19) with the STAR aligner (v2.5.0a)60. Gene count quantification for total RNA was performed using the GeneCounts function within STAR against the GENCODE v19 transcript file.

Gene count data were input into edgeR software61 for differential expression analysis. Briefly, gene counts were normalized against total aligned reads for each sample to generate counts per million (cpm) expression value for each gene in each sample. Given the relatively small sample size per group for comparison ($n = 3$ in each group) the exact test implemented in edgeR was used to determine differential expression including a false discovery rate *P*-value.

To discover enriched functional-related gene groups, bioinformatics Database for Annotation, Visualization, and Integrated Discovery (DAVID) was used (NIH, https://davidbioinformatics.nih.gov/).

Protein extraction and immunoblotting

Podocytes or tissue from kidney cortices, glomeruli or tubular fractions of mice were homogenized in ice-cold CHAPS buffer supplemented with protease and phosphatase inhibitor cocktails. Protein concentration was quantified using the Pierce™ BCA Protein Assay kit according to the manufacturer's protocol. Samples were prepared in CHAPS buffer supplemented with 0.1% SDS Laemmli buffer. At least 15 μg of protein was loaded onto 4-20% SDS-PAGE gels followed by transfer onto PVDF membranes. Membranes were blocked in 3% BSA for phospho-proteins and in 5% skim milk for total proteins at 4°C, overnight followed by incubation with primary antibodies at 4°C, overnight. Blots were scanned using the Azure Biosystems C600 imaging system (Dublin, CA, USA). Antibodies used in the study and their respective dilutions are summarized in Supplementary Table 2. Uncropped blot images are shown in Supplementary Figure S9.

Cell death analysis

Apoptosis was assessed using the Caspase-3 Apo-Tox-Glow Triplex Assay (Promega) according to the manufacturer's instructions. Briefly, differentiated podocytes were treated with S1P (0.1, 0.2, 1, 5 and 20 μM) or C1P (0.1, 0.2, 1, 5 and 20 μM) for 24 h and Caspase-3 activity was determined after 2 h at excitation 470/ emission 520 for the former, or excitation 400/ emission 505 for viability and luminescence for 1 sec for Caspase-3 activity for the latter. Values are expressed as fold change to controls.

Liquid Chromatography Mass Spectrometry (LC-MS) Analysis

Kidney tissue from mice was homogenized by brief sonication and lysed in RIPA buffer for 30 min on ice followed by centrifugation at +4°C, 14,000 rpm for 15 min. Protein concentration was measured using BCA kit (Thermo Fisher Scientific) and kidney tissue samples containing 1 mg of protein were used for

LC-MS analysis. For murine podocytes in culture, cell pellets containing at least $1x10^6$ cells per sample were used for LC-MS analysis. LC-MS analysis was performed in Medical University of South Carolina, Lipidomics shared resource (SC, USA). Cell pellets and kidney tissue were subjected to liquid extraction as reported previously[5]. For the detection of sphingolipids in urine, 50-200 μl of urine from mice were utilized for LC-MS analysis. The levels of sphingolipids (pmol) were normalized to milliliters (mL) of urine, and the ratio over urine creatinine levels (mg/mL) was utilized to present the final graphs.

Matrix-assisted laser desorption ionization - mass spectrometry imaging (MALDI-MSI)

Three mice per group (CTRL, Col4a3$^{-/-}$, DKO) were used. Cryo-sectioned kidney cortex tissue sections (10 μm) from mice were mounted on indium tin oxide (ITO) coated glass slides (Bruker Daltonics, Germany). Subsequently, the slides were subjected to vacuum desiccation for 30 min prior to matrix application. A multimodal imaging approach was adopted to explore the spatial distribution of metabolites/lipids within glomeruli of the tissue sections. Bright-field (BF) microscopy facilitated the visualization of glomerular morphology and other pathological features. Autofluorescence (AF) microscopy (ZEISS Axioscan 7 Microscope Slide Scanner (Oberkochen, Germany) aided in delineating the regions corresponding to glomeruli. Additionally, PAS staining of serial sections was conducted to validate the localization of glomeruli within the tissue. Optical images generated were subsequently uploaded to METASPACE and SCiLS Lab software for data analysis, enabling the overlay of metabolite/lipid images with optical images. Statistical analysis was performed utilizing data from the entire tissue section, with specific regions of interests (ROIs), such as glomeruli, compared for the levels of S1P species.

For lipid molecule imaging, both negative and positive ion modes were employed to encompass the spectrum of S1P species. In the negative ion mode, a solution of 1,5-Diaminonaphthalene matrix (DAN; 5.55 mg/mL in 50% ethanol:H_2O +5% HCl) was utilized, with the following spraying parameters: nozzle temperature of 80 °C, a flow rate of 0.025 mL/min, 20 passes, a nitrogen pressure of 10 psi, a track spacing of 2 mm. The distance between the nozzle and sample was consistently maintained at 40 mm during the samples preparation. Conversely, in the positive ion mode, 2,5-dihydroxybenzoic acid (DHB; 40 mg/mL in 50% methanol:H_2O) was applied to the tissue section. Spraying parameters include a nozzle temperature of 80 °C, a flow rate of 0.05 mL/min, 10 passes, a N^2 pressure of 10 psi, a track spacing of 3 mm, and a 40 mm distance between the nozzle and sample was maintained for preparation of all samples.

MSI images were acquired using a Q Exactive HF-X hybrid quadrupole-Orbitrap mass spectrometer (Thermo Scientific, Emeryville, CA, USA) in combination with an innovative elevated pressure MALDI/ESI interface (Spectroglyph LLC, Kennewick, WA, USA)[6]. Mass spectra were obtained in the mass range of *m/z* 100-1,000. The Spectroglyph MALDI Injector Software facilitated the control of raster step size over the tissue region to be imaged. To generate images, the spectra were collected at 20 μm intervals in both

the X and Y dimensions across the surface of the sample. MSI data were acquired using an Orbitrap instrument set to a nominal mass resolution of 120,000, a 200 ms ion injection time, and automatic gain control disabled. Ion images were constructed from raw files (obtained from Orbitrap tune software) and position files (obtained from MALDI Injector Software) using Image Insight software (Spectroglyph, LLC). The centroid and profile data were exported into the imzML and ibd formats using Image Insight and SCiLS Lab software version 2020c Pro (SCiLS, Bremen, Germany) separately[7].

Sample preparation for Atomic Force Microscopy (AFM)

At the time of sacrifice, a portion of one mouse kidney was embedded in cryomatrix and stored at −80°C. Cryosectioning of both cryomatrix-embedded mouse kidney tissue and human kidney biopsy samples was performed at −20°C using a Leica CM3050S cryostat (Leica Biosystems Canada, Concord, Ontario, Canada). Longitudinal kidney sections (10 µm thick) were collected on SuperFrost Plus glass slides (Globe Scientific Inc., Mahwah, NJ) and stored at −80°C until further processing. Before staining, the sections were dried for 30 minutes at room temperature and then lightly fixed with 4% paraformaldehyde for 8 minutes at room temperature to ensure adherence to the slides.

Picrosirius red (PSR) staining was performed following the protocol described previously[8]. Kidney sections were immersed in xylene twice for 10 minutes at room temperature and then sequentially rehydrated through decreasing concentrations of ethanol (100%, 96%, and 70% ethanol, 10 seconds each). Sections were stained with a 0.1% solution of Sirius Red F3BA in a saturated aqueous solution of picric acid for 1 hour at 25°C. After staining, slides were washed twice in 0.5% acetic acid, dehydrated in increasing concentrations of ethanol (70%, 96%, and 100%, 10 seconds each), and cleared with two changes of xylene for 10 minutes each.

Silver staining was carried out by immersing kidney sections in 0.5% periodic acid for 15 minutes, followed by multiple washes in distilled water. Sections were incubated in a preheated working silver solution (3% hexamine, 5% silver nitrate, and 5% sodium tetraborate) in a hot air oven at 65–72°C for 40 minutes, with periodic agitation. After 20 minutes, the slides were examined microscopically to assess the degree of silver impregnation of the glomeruli. The sections were then rinsed in distilled water, treated with 0.2% gold chloride for 2 minutes, and washed multiple times in distilled water. Finally, the slides were placed in 5% sodium thiosulfate for 3 minutes, followed by additional washes in distilled water.

Polyclonal anti-Wilms Tumor Protein antibody-stained sections (were used for podocyte stiffness analysis.

Atomic Force Microscopy (AFM) measurement conditions

To evaluate tissue stiffness, nano-indentation measurements were performed using atomic force microscopy (AFM) on stained kidney sections. All AFM measurements were performed at room temperature in a liquid environment using a Bruker Resolve AFM system (Bruker, USA) integrated with

an Olympus IX-83 inverted optical microscope. The measurements were conducted with different probe types. Specifically, spherical silicon dioxide (SiO_2) tips measuring 20 µm and 5 µm in diameter were affixed to a pre-calibrated silicon nitride cantilever (spring constant: 0.038 N/m; nominal resonance frequency: 10 kHz, Novascan Technologies Inc., Ames, Iowa, USA) for assessing glomerular and podocyte stiffness, respectively, as previously described. The stiffness of the glomerular basement membrane (GBM) was determined using a sharp AFM tip (spring constant: 0.03 N/m; nominal resonance frequency: 10 kHz, HQ:CSC38/B, MikroMasch Co., Tallinn, Estonia). Force spectroscopy was conducted by detecting cantilever deflection and z-piezo movement at each indentation step, generating force-displacement curves based on the known cantilever spring constant. The indentation speed was set between 2.5 µm/s and 10 µm/s, with a maximum indentation depth of 500 nm. Stiffness values were calculated using a Hertzian model of surface indentation. Nano-indentation experiments were conducted by applying the spherical AFM tip at the center of each glomerulus to measure glomerular stiffness and directly over the podocyte nucleus to evaluate podocyte stiffness. The sharp AFM tip was used to assess GBM stiffness. Stiffness values were obtained from five podocytes per glomerulus (30 glomeruli per section) and at four standardized locations along the GBM (12, 3, 6, and 9 o'clock positions) in 30 glomeruli per section.

Mice

The animals were housed in the animal facility of the Division of Veterinary Resources, University of Miami, Miller School of Medicine, on 12-h light/dark cycles under controlled temperature (22±1°C) and provided water and Teklad Global 18% protein rodent chow diet (Alice, TX, USA) ad libitum. Both the research team and the veterinary staff monitored animals daily. Health was monitored by weight (bi-weekly), food and water intake (daily), and general assessment of animal activity, panting, and fur condition (daily). Any signs of illness were logged as "adverse events" in the experiment, the mouse was immediately killed. The mice were euthanized using ketamine/xylazine mix in 0.9% NaCl. After perfusion of an animal with 1xPBS, the right kidney was removed for histological analyses and the left kidney was harvested for glomeruli isolation.

No inclusion or exclusion criteria were used to assign animals into groups.

Generation of Col4a3 knockout mice with podocyte specific Smpdl3b deficiency (DKO)

Col4a3 heterozygous ($Col4a3^{+/-}$) mice were purchased in a 129X1/SvJ background (129-$Col4a3^{tm1Dec}$/J, #002908, Jackson Laboratories, Bar Harbor, ME, USA) and backcrossed to C57BL6/J mice (#000664, Jackson Laboratories) for 10 generation. Then, $Col4a3^{+/-}$ littermates were bred to generate homozygous Col4a3 KO mice in a C57BL6 background ($Col4a3^{-/-}$). $Col4a3^{-/-}$ mice were bred to homozygous podocyte-specific *Smpdl3b* deficient mice ($Smp^{-/-}$)[9] to obtain double heterozygous mice. Finally, the latter were intercrossed to generate homozygous $Col4a3^{-/-}$ mice with podocyte specific

homozygous *Smpdl3b* deficiency (DKO). Wildtype littermates (CTRL) and $Smp^{-/-}$ mice were used as controls. A detailed scheme for the generation of DKO mice is shown in Supplementary Figure S1a.

For genotyping, tissues from tail biopsies of 3-week-old mice were digested with proteinase K and DNA was isolated using QIAamp DNA Mini kit according to the manufacture's protocol. Podocin-Cre-recombinase alleles and absence of Smpdl3b alleles, which are characterized by the presence of a 3'-loxP site downstream of exon 2 of the *Smpdl3b* gene, were detected using PCR as described previously[9]. The Col4a3 KO allele was detected by PCR using the genotyping protocol provided by the manufacturer. Primer sequences used for the gene are listed in Supplementary Table 1. A representative genotyping PCR is shown at the Supplementary Figure S1b.

Starting at 4 weeks of age, weight measurements and morning spot urines were collected bi-weekly until mice reached 20 weeks of age. Male and female mice were analyzed in all study groups. Four groups of mice were used in the study: 1) wildtype control mice (CTRL, n=11); 2) mice with podocyte specific *Smpdl3b* deletion ($Smp^{-/-}$, n=11); 3) Col4a3 KO mice ($Col4a3^{-/-}$, n=11); 4) Col4a3 KO mice with podocyte specific *Smpdl3b* deletion (DKO, n=11).

Generation of mice with doxycycline-inducible podocyte specific Smpdl3b overexpression

To generate mice with a doxycycline-inducible podocyte specific *Smpdl3b* overexpression, a Myc-DDK-SMPDL3b cDNA was utilized to amplify *Smpdl3b* using specific primers (Supplementary Table 1) followed by separation using gel electrophoresis. The band corresponding to the full-length cDNA of *Smpdl3b* was excised, blunted and purified using the Gel Extraction and PCR Purification kits and then cloned into the pTRE3G vector, which contains an inducible Tet-responsive element. To generate an Smpdl3b inducible vector Myc-DDK-SMPDL3b-pTRE3G, the blunted cDNA product was cloned into a blunted vector and TetO-Myc-DDK-SMPDL3b insert was released from the vector backbone using AseI and utilized for pronuclear injection in fertilized oocytes of C57BL/6 mice to generate *Smpdl3b* transgenic mice (SMP^{Tg}). To generate mice with podocyte specific doxycycline inducible Myc-DDK-SMPDL3b overexpression ($pSMP^{Tg}$), SMP^{Tg} founders were bred to podocin-rtTA mice[10] (Supplementary Figure S2a). Podocyte Myc-DDK-SMPDL3b expression was induced by feeding the mice 2000 ppm doxycycline containing chow for 30 days, starting at 28 days of age.

For genotyping, tissues from tail biopsies of 3-week-old mice were digested with proteinase K and DNA was isolated using QIAamp DNA Mini kit according to the manufacture's protocol. Presence of *rtTA* allele and *Smpdl3b* allele was detected by PCR using the specific primers as listed in Supplementary Table 1. rtTA mice were identified using specific primers for this transgene, yielding a 455 bp PCR product, and $pSMP^{Tg}$ mice were identified using specific primers for *Smpdl3b* transgene, yielding a 300 bp PCR product (Supplementary Figure S2b). Starting at 4 weeks of age, weight measurements and morning spot urines were collected monthly until mice reached 32 weeks of age. Male and female mice were analyzed in all study groups: 1) $pSMP^{WT}$ Dox+ (wildtype mice on doxycycline diet), n=7; 2) $pSMP^{Tg}$

Dox+ (mice with doxycycline-induced podocyte-specific *Smpdl3b* overexpression), n=9 and 3) $pSMP^{Tg}$ Dox- (pSMP transgenic mice on normal chow in which podocyte-specific *Smpdl3b* overexpression was not induced), n=8.

Sphingosine-1-phosphate (S1P)/albumin treatment of mice

Mice were treated with S1P (d17:1), which is not endogenous and more stable form than other S1P species thus allowing for better tissue tracking over time and quantification of endogenous S1P (d18:1). S1P (d17:1) powder was solubilized in heated DMSO solution (2.6 mM) followed by dilution in 4 mg/mL fatty acid free albumin solution to prepare the final solution for the treatment. DMSO dissolved in 4 mg/mL fatty acid free albumin solution was used as vehicle control. 100 μl of S1P (100 nM or 0.0002 mg/kg) or DMSO (5%) were injected interperitoneally daily for 4 weeks. 16-week-old male and female mice were randomly divided into six groups: 1) CTRL DMSO (n=5); 2) CTRL S1P (n=7); 3) $Col4a3^{-/-}$ DMSO (n=5); 4) $Col4a3^{-/-}$ S1P (n=7); 5) DKO DMSO (n=5); 6) DKO S1P (n=7).

Urine sample analysis

The urinary albumin content was measured by sandwich ELISA following the manufacturer's protocol. The urinary creatinine was measured by an assay based on the Jaffe method, using albumin ELISA kit and creatinine kit. Values are expressed as microgram of albumin per milligram of creatinine.

Blood sample analysis

Blood samples were analyzed for blood urea nitrogen (BUN) in the Comparative Laboratory Core Facility of the University of Miami. Serum creatinine was determined by tandem mass spectrometry at the UAB-UCSD O'Brien Core Center (University of Alabama, Birmingham, AL) as it was previously described [11].

Glomerular filtration rate (GFR) measurement

GFR was measured using intravenous injections with FITC-sinistrin (7.5 mg/100 g body weight) as described previously [12]. Briefly, FITC-sinistrin was dissolved in 0.9% saline solution and intravenously injected at the dose of 7.5 mg/ 100 g body weight into previously anesthetized (5% isoflurane at 5 L/min oxygen flow) mice. The fur was removed from the flank of the back of an animal and the optical device to measure the renal function had been placed using double-sided adhesive patch. The background was measured for 1 min before FITC-sinistrin injection. The fluorescence emitted by FITC-sinistrin was measured for 1 h after injection. Then the device was removed and connected to a PC to download the data. Using the software provided, the elimination kinetics curve was generated. For the evaluation of the data, the background signal measured prior FITC-sinistrin administration was set and the beginning of the exponential excretion phase of the marker was marked (which usually occurred 15 min after the

injection). The software automatically displayed the FITC-sinistrin half-life ($t_{1/2}$) along with an R^2 value, which is determined by a 1-compartment model. $t_{1/2}$ can be utilized to calculate GFR by using a conversion factor[12].

Glomeruli isolation

Glomeruli were isolated from mouse kidneys using a microdissection technique. Kidneys were perfused with ice-cold 1xPBS and minced into small pieces, and the left kidney was harvested and mashed in 1xPBS buffer and processed through sieving steps using 100 μm and 70 μm Falcon sieve strainers. The glomeruli-enriched fraction was collected by centrifugation at 3,000×g for 5 minutes and washed twice with ice-cold PBS. The isolated glomeruli were resuspended in appropriate buffer or media for further analysis.

Periodic acid-Schiff (PAS) staining and assessment of mesangial expansion

PAS staining of paraffin-embedded kidney sections (4 μm thick) was performed using a standard protocol. Kidney sections were deparaffinized in xylene and rehydrated through a graded ethanol series. Slides were incubated in 0.5% periodic acid solution for 10 minutes at room temperature, followed by rinsing in distilled water. Sections were then stained with Schiff's reagent for 15 minutes, washed in running tap water for 5 minutes, and counterstained with hematoxylin for 1 minute. After washing, slides were dehydrated through graded ethanol, cleared in xylene, and mounted with a permanent mounting medium. Histological images were visualized using a light microscope (Olympus BX 41, Tokyo, Japan) at x40 magnification and analyzed using Image J software [13]. Sixty glomeruli per section were analyzed for mesangial expansion by semi quantitative analysis (scale 0–4) performed by two blinded independent investigators [14, 15].

Picrosirius Red (PSR) staining and assessment of fibrosis

Kidney sections (3–5 µm) were deparaffinized in xylene and rehydrated through a graded ethanol series. Slides were incubated in Picrosirius Red solution (0.1% Sirius Red in saturated picric acid) for 60 minutes at room temperature. Excess stain was removed by rinsing twice in 0.5% acetic acid. Sections were then dehydrated through graded ethanol, cleared in xylene, and mounted with a permanent mounting medium. Histological images were visualized using a light microscope (Olympus BX41, Tokyo, Japan) at x40 magnification and analyzed using Image J software [16]. High-resolution images were acquired under polarized light. Fibrotic areas were quantified by applying color thresholding to isolate PSR-positive regions. The percentage of fibrosis was calculated as the collagen-stained area relative to the total tissue area.

Anti-Wilms' tumor 1 (WT1) staining

Kidney sections (4 µm) were deparaffinized, rehydrated, and subjected to antigen retrieval using citrate buffer (pH 6.0) at 110°C for 10 minutes. Permeabilization was performed using 0.3% Triton X-100 in 1xPBS for 10 min at room temperature. A blocking step was performed using 5% BSA, 2.5% FBS in PBS for 1 h at room temperature. After blocking, sections were incubated overnight at 4°C with anti-WT1 primary antibody (1:300). The next day, sections were incubated with Alexa fluorophore-conjugated secondary antibody (1:500) for 1 hour at room temperature. Slides were washed in 1xPBS, dehydrated, and mounted. Histological images were visualized using a Leica SP5 Inverted microscope, x40 wet objective (Leica Microsystems CMS GmbH, Mannheim, Germany). WT1-positive podocytes were counted in glomeruli using ImageJ by two blinded investigators[17].

Immunoperoxidase staining

Paraffin-embedded kidney sections (4 μm) from human biopsies were used. Prior deparaffinization, slides were baked at 60°C for 1 h. Deparaffinization was performed in 100% xylene for 5 min (3 changes) followed by rehydration in graded alcohol series (5 min incubation in 100%, 95%, 70% and 50% ethanol, 1 change each) and 5 min incubation in deionized water (1 change). Antigen retrieval step was not applied. Slides were incubated with hydrogen peroxidase blocking solution for 10 min at room temperature followed by protein block for 1 h at room temperature. Anti-SMPDL3b antibodies were applied in 1:100 dilution overnight at room temperature followed by incubation with Goat anti-Rabbit IgG H&L antibodies. DAB substrate was added to the sections for 5 min at room temperature followed by counterstain with hematoxylin (for 7 min at room temperature. Histological images were visualized using a light microscope (Olympus BX 41, Tokyo, Japan) at x20 magnification.

Synaptopodin staining and fluorescence measurement

Kidney sections (4 µm) were deparaffinized, rehydrated, and subjected to antigen retrieval using citrate buffer (pH 6.0) at 110°C for 10 minutes. Permeabilization was performed using 0.3% Triton X-100 in 1xPBS for 10 min at room temperature. A blocking step was performed using 5% BSA, 2.5% FBS in PBS for 1 h at room temperature. After blocking, sections were incubated overnight at 4°C with anti-WT1 primary antibody (1:300). The next day, sections were incubated with Alexa fluorophore-conjugated secondary antibody (1:500) for 1 hour at room temperature. Slides were washed in 1xPBS, dehydrated, and mounted. Histological images were visualized using a Leica SP5 Inverted microscope, x40 wet objective (Leica Microsystems CMS GmbH, Mannheim, Germany). Fluorescent images were acquired by laser scanning confocal microscopy using a Leica SP5 Inverted microscope, 40x wet objective (Leica Microsystems CMS GmbH, Mannheim, Germany). Measuring of cell fluorescence was performed using Image J software as described previsouly[18]. Corrected total cell fluorescence (CTCF) was calculated using formula:

CTCF = Integrated Density – (Area of selected cell x Mean fluorescence of background readings).

TUNEL assay

TUNEL (Terminal deoxynucleotidyl transferase dUTP nick end labeling) assay was carried out to the manufacturer's protocol. Briefly, after deparaffinization, rehydration and antigen retrieval steps as described above, tissue slides were incubated in 20 µg/mL Proteinase K solution (Tris-HCl pH 8.0, 50 mM EDTA) for 5 min at room temperature. 100 µl of DNA labeling solution were applied to each slide and slides were incubated for 1 h at 37°C. The slides were then washed with PBS followed by the incubation with anti-BrdU-Red antibodies (2.5 µl per reaction) for 30 min at room temperature. DNA was counterstained using 7-AAD/RNase A staining buffer for 30 min at room temperature. Images were acquired by high-throughput VS120 Olympus slides scanner, x4-x20 air lenses, 4 channels: DAPI, FITC, TRITC, Cy5. The number of TUNEL-positive cells in glomeruli was counted using high-resolution images of whole kidney section, and the results were expressed as the number of positive cells per glomerulus.

Transmission electron microscopy (TEM)

For ultrastructural analyses, samples were fixed in 2% paraformaldehyde/2.5% glutaraldehyde (Polysciences Inc., Warrington, PA) in 100 mM sodium cacodylate buffer, pH 7.2 for 2 h at room temperature and then overnight at 4°C. Samples were washed in sodium cacodylate buffer at room temperature and postfixed in 1% osmium tetroxide (Polysciences Inc.) for 1 h. Samples were then rinsed extensively in dH_2O prior to en bloc staining with 1% aqueous uranyl acetate (Ted Pella Inc., Redding, CA) for 1 h. Following several rinses in dH20, samples were dehydrated in a graded series of ethanol and embedded in Eponate 12 resin (Ted Pella Inc.). Sections of 95 nm were cut with a Leica Ultracut UCT ultramicrotome (Leica Microsystems Inc., Bannockburn, IL), stained with uranyl acetate and lead citrate, and viewed on a JEOL 1200 EX transmission electron microscope (JEOL USA Inc., Peabody, MA) equipped with an AMT 8-megapixel digital camera and AMT Image Capture Engine V602 software (Advanced Microscopy Techniques, Woburn, MA).

Statistical analysis and study design

Data are expressed as a mean ± standard deviation (SD). A number of experiments ranging between 3 and 5 was utilized and as indicated for each distinct experiment. Investigators were blinded for imaging and data analyses. No sample size calculations were performed for *in vitro* studies. Minimal group sizes for *in vivo* studies were determined via power calculator using G Power (https://www.psychologie.hhu.de/arbeitsgruppen/allgemeine-psychologie-und-arbeitspsychologie/gpower.html) with an α of 0.05 and effect size (d) of 2 (using guidelines from Ref.[19]). Statistical analyses were performed using the GraphPad Prism, version 9.0 (GraphPad Software Inc.).

Data were tested for normality using the Kolmogorov-Smirnov or Shapiro-Wilk test, and equality of variance was confirmed using the F-test. Two groups of data were compared using the one-tailed unpaired Student's t-test. Three and more groups of data were compared using the one-way analysis of

variance (ANOVA) or two-way ANOVA, followed by Tukey’s post hoc tests. $p<0.05$ was taken to indicate statistical significance. Simple randomization for the animal groups was used to generate the randomization sequence using Research Randomizer tool (https://www.randomizer.org/). Confounders were not controlled. All data points were included into analyses.

For MALDI-MSI, regions of interest (ROIs) such as glomeruli were localized based on AF/BF images and further confirmed based PAS images from serial sections. S1P profiles were extracted from whole kidney tissue sections of CTRL, Col4a3$^{-/-}$, and DKO mice (n=3 mice/group) for statistical comparisons and further confirmed in glomeruli based on overlayed MALDI-MSI ion images and AF/PAS images. Multivariate analyses were performed for S1P lipids in the whole kidney tissue sections of three groups of mice. Principal component analysis (PCA), as an unsupervised statistical method, was used to detect outliers and find the structure of the data. One-way ANOVA was used for comparisons of individual S1P species in kidneys of three groups of mice. Metabolite identification was done for the annotations extracted from HMDB and SwissLipids databases in METESPACE (which were further confirmed by LC-MS/MS analysis).

SUPPLEMENTARY FIGURES

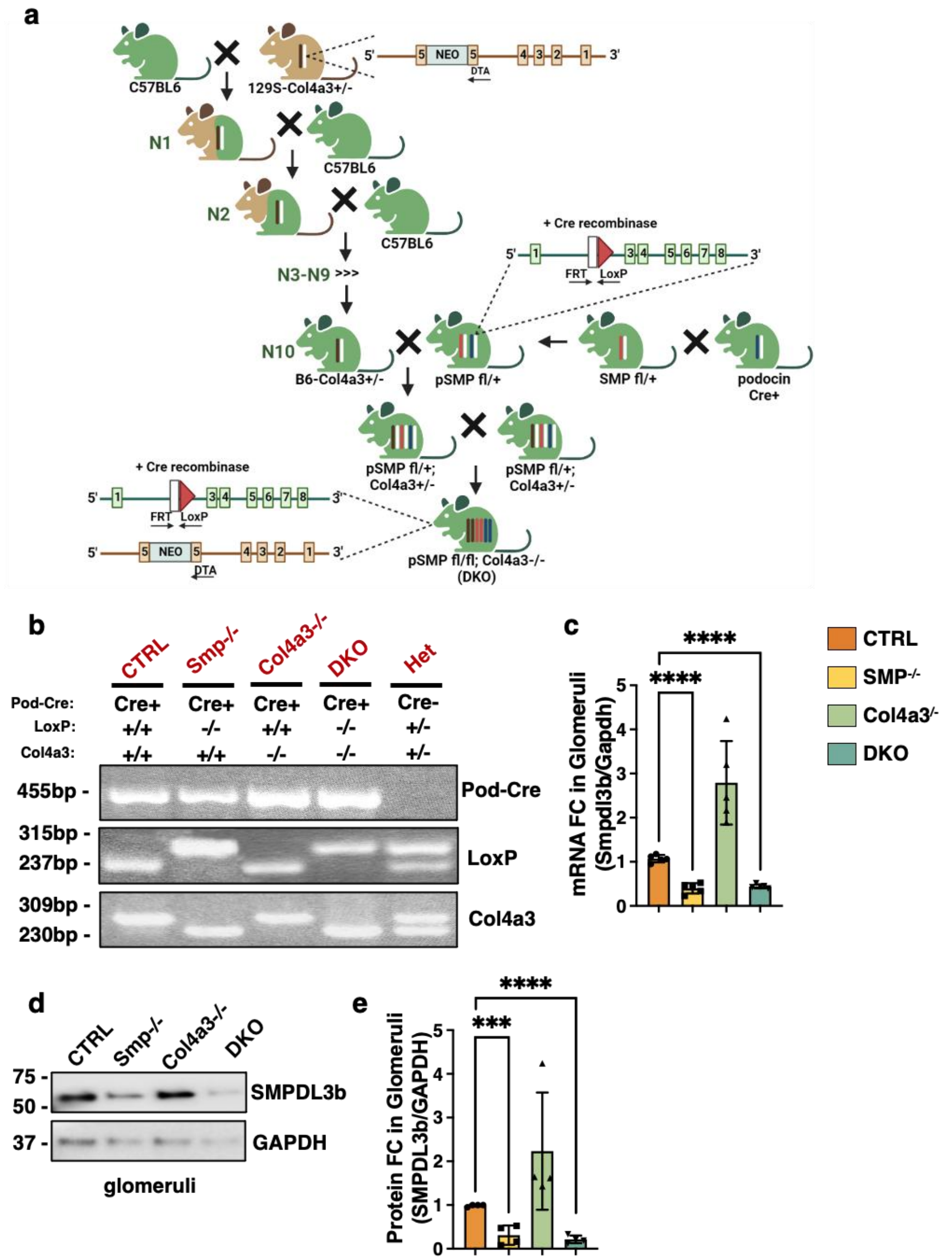

Supplementary Figure S1. Generation, genotyping PCR and verification of the efficacy of producing Col4a3$^{-/-}$ with podocyte specific *Smpdl3b* deficiency. **(a)** Schematic representation of the generation of Col4a3$^{-/-}$ with podocyte specific *Smpdl3b* deficiency (DKO). **(b)** PCR on genomic DNA isolated from tails biopsy showing amplification products of a 455bp band in mice expressing the Podocin-Cre (Pod-Cre) transgene (Cre+), of a single 315bp band in mice carrying only *Smpdl3b*$^{+/+}$ (wildtype) alleles, of a 237bp band in mice a carrying only *Smpdl3b* floxed alleles (SMP-/-) and both bands in heterozygous (SMP+/-, Het). Similarly, a single 309bp band was detected in mice carrying only the Col4a3 wildtype allele in Col4a3$^{+/+}$, a 230bp band in mice carrying only the Col4a3 knockout allele (Col4a3$^{-/-}$) and both bands in heterozygous (Het) mice. **(c)** mRNA isolated from glomeruli of wildtype mice (CTRL, n=4), mice with podocyte specific *Smpdl3b* deletion (Smp$^{-/-}$, n=4), Col4a3$^{-/-}$ mice (n=4) and Col4a3$^{-/-}$ mice with podocyte specific *Smpdl3b* deletion (DKO, n=4) was used and Smpdl3b expression was analyzed in RT cDNA by qRT-PCR. **(d-e)** Representative Western blot **(d)** and bar graph analysis

(e) of SMPDL3b expression in glomeruli isolated from CTRL, Smp$^{-/-}$, Col4a3$^{-/-}$ and DKO mice. GAPDH served as a loading control. Data are mean±SD. *P* values were calculated using One-Way ANOVA.

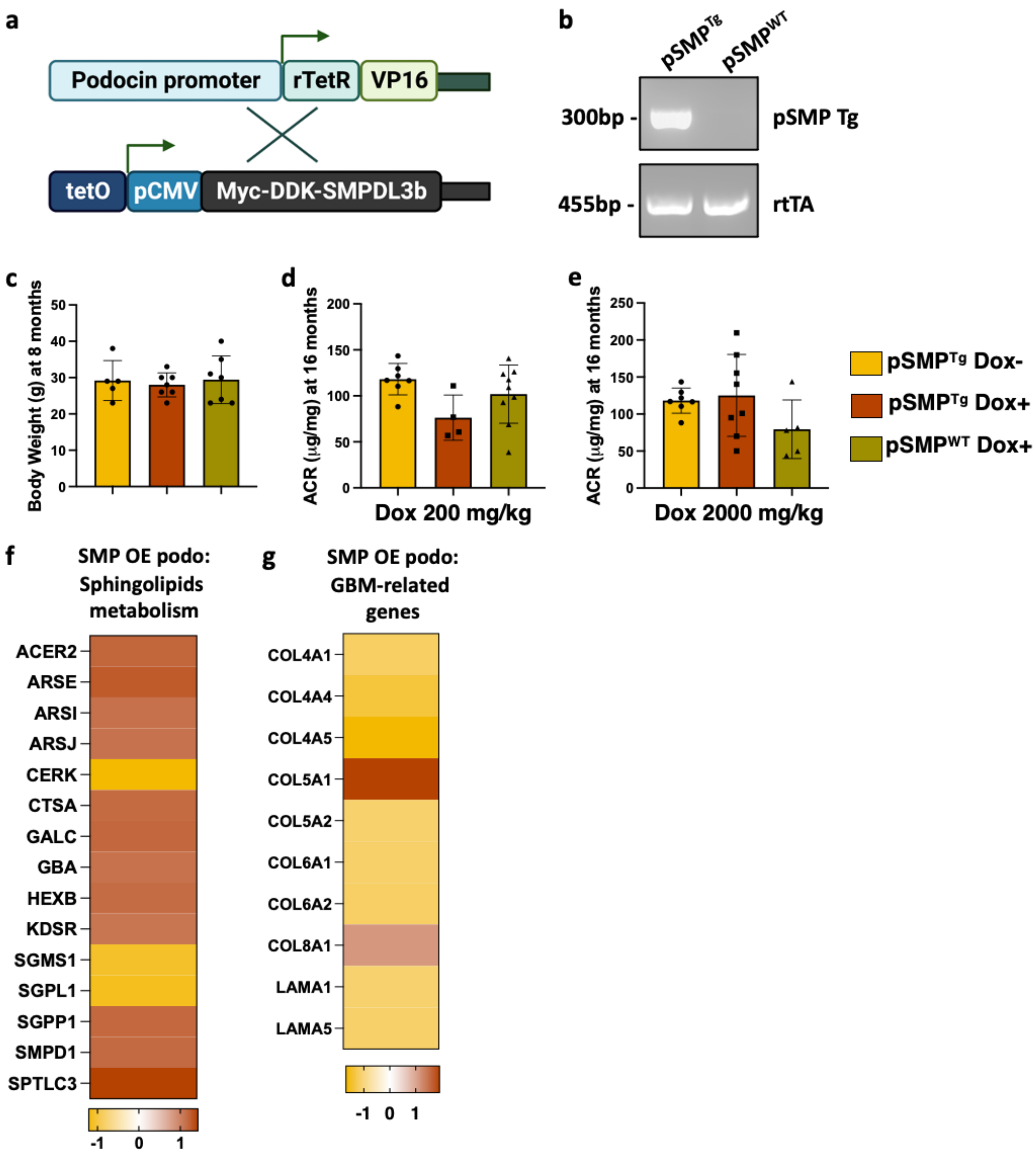

Supplementary Figure S2. Generation, genotyping PCR and extended data related to mice with doxycycline-induced podocyte specific *Smpdl3b* overexpression. **(a)** Schematic representation of the generation of mice with doxycycline-inducible podocyte specific *Smpdl3b* overexpression. **(b)** PCR on genomic DNA isolated from tails biopsy showing amplification products of 455bp in mice expressing the Podocin-rtTA (rtTA) transgene and a 300 bp band in Smpdl3b transgenic mice (pSMP^{Tg}). **(c)** Body weight in 32-week-old mice from uninduced pSMP Tg mice (pSMP^{Tg} Dox-), induced pSMP^{Tg} mice (pSMP^{Tg} Dox+) and rtTA control (pSMP^{WT} Dox-) groups. **(c)** Body weight at 8-month-old animals used in the study: 1) uninducible mice with podocyte specific *Smpdl3b* overexpression (pSMP^{Tg} Dox-, n=5), 2) doxycycline-inducible mice with podocyte specific *Smpdl3b* overexpression (pSMP^{Tg} Dox+, n=7), and 3) doxycycline-inducible mice with intact Smpdl3b expression in podocytes (pSMP^{WT} Dox+, n=7). **(d-e)** Albumin-to-creatinine ratio (ACR) in all three experimental groups of mice fed on doxycycline 200 **(d)** and 2,000 **(e)** diet. **(f-g)** Bulk RNA-seq analysis of immortalized human podocytes with SMPDL3b overexpression showing changes in sphingolipid metabolism genes **(f)** and glomerular basement membrane (GBM)-related genes **(g)**. Genes that passed false discovery rate (FDR) correction (q≤0.05)

for multiple testing were considered as significantly regulated and are highlighted in red (increased expression) or yellow (decreased expression).

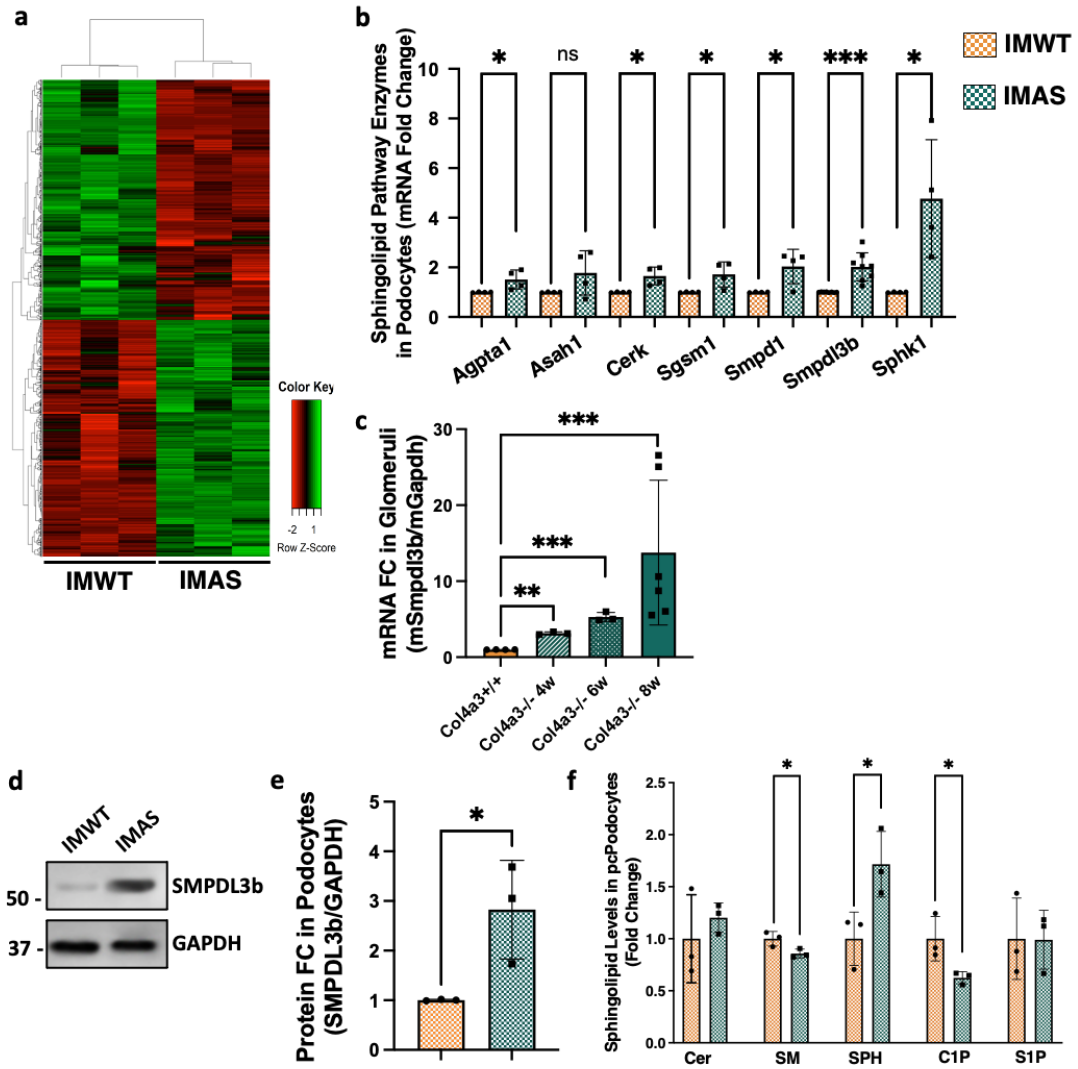

Supplementary Figure S3. Immortalized mouse podocytes isolated from Col4a3$^{-/-}$ mice have increased expression of SMPDL3b in association with dysregulated sphingolipid metabolism. (a) Heat map showing differential genes expression in immortalized murine podocytes isolated from control (IMWT) and Col4a3$^{-/-}$ (IMAS) mice. Increased gene expression is highlighted in green and decreased gene expression is highlighted in red (GSE274298). **(b)** qRT-PCR analysis of mRNA expression of enzymes regulating sphingolipid metabolism in IMWT and IMAS immortalized murine podocytes. **(c)** qRT-PCR analysis of *Smpdl3b* mRNA expression in glomeruli isolated from Col4a3$^{+/+}$ at 8 weeks of age and Col4a3$^{-/-}$ mice at 4, 6 and 8 weeks of age. **(d-e)** Representative Western blot **(d)** and bar graph analysis **(e)** of SMPDL3b expression in IMWT and IMAS murine podocytes. n=3 independent experiments. **(f)** LC-MS analysis of total ceramide (Cer), sphingomyelin (SM), sphingosine (SPH), ceramide-1-phosphate (C1P) and sphingopsine-1-phosphate (S1P) levels in IMWT and IMAS murine podocytes. n=3 independent experiments. Data are mean±SD. *P* values were calculated using two-tailed t-test.

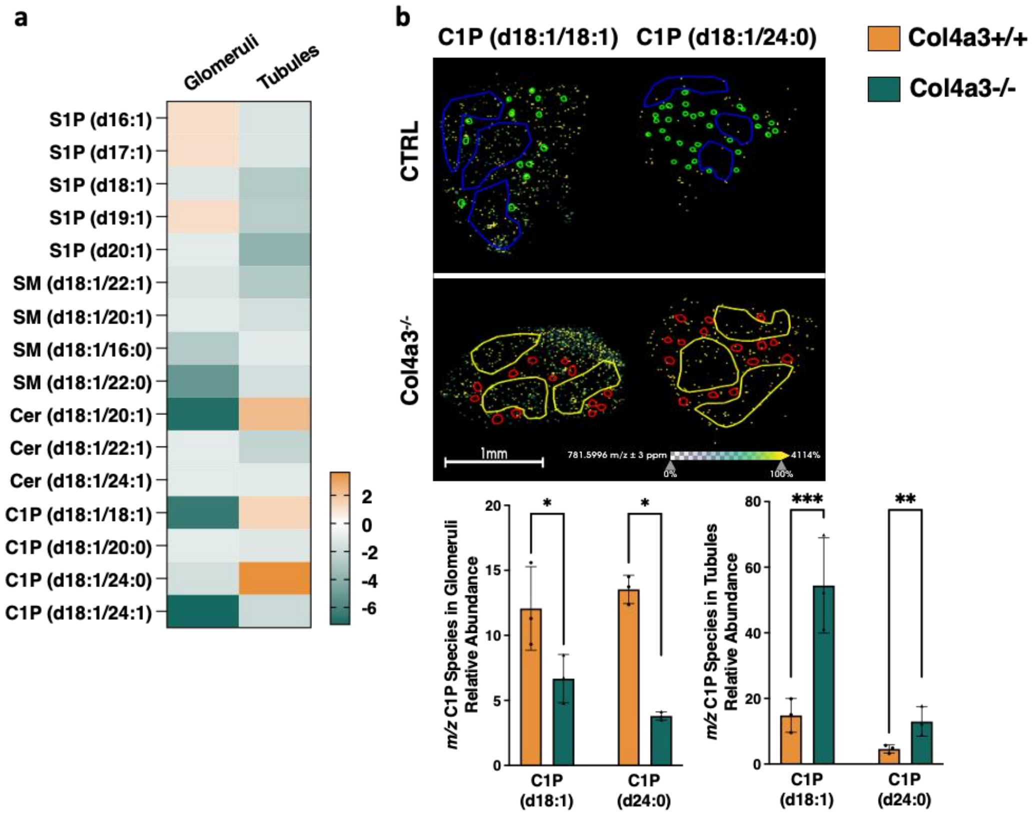

Supplementary Figure S4. Spatial metabolomic of sphingolipid species in glomeruli and tubular region isolated from Col4a3$^{-/-}$ mice. (a) Heat map of sphingomyelin (SM), sphingosine-1-phosphate (S1P), ceramide (Cer) and ceramide-1-phosphate (S1P) species in glomeruli and tubules in Col4a3$^{-/-}$ mice. Increased levels are highlighted in orange and decreased levels are highlighted in green. **(b)** Overlayed AF images (top) and related bar graph analysis (bottom) of C1P species detected in glomeruli (green and red for Col4a3$^{+/+}$ and Col4a3$^{-/-}$ mice, respectively) and tubules (blue and yellow for Col4a3$^{+/+}$ and Col4a3$^{-/-}$ mice, respectively). Data are mean±SD. *P* values were calculated using two-tailed t-test.

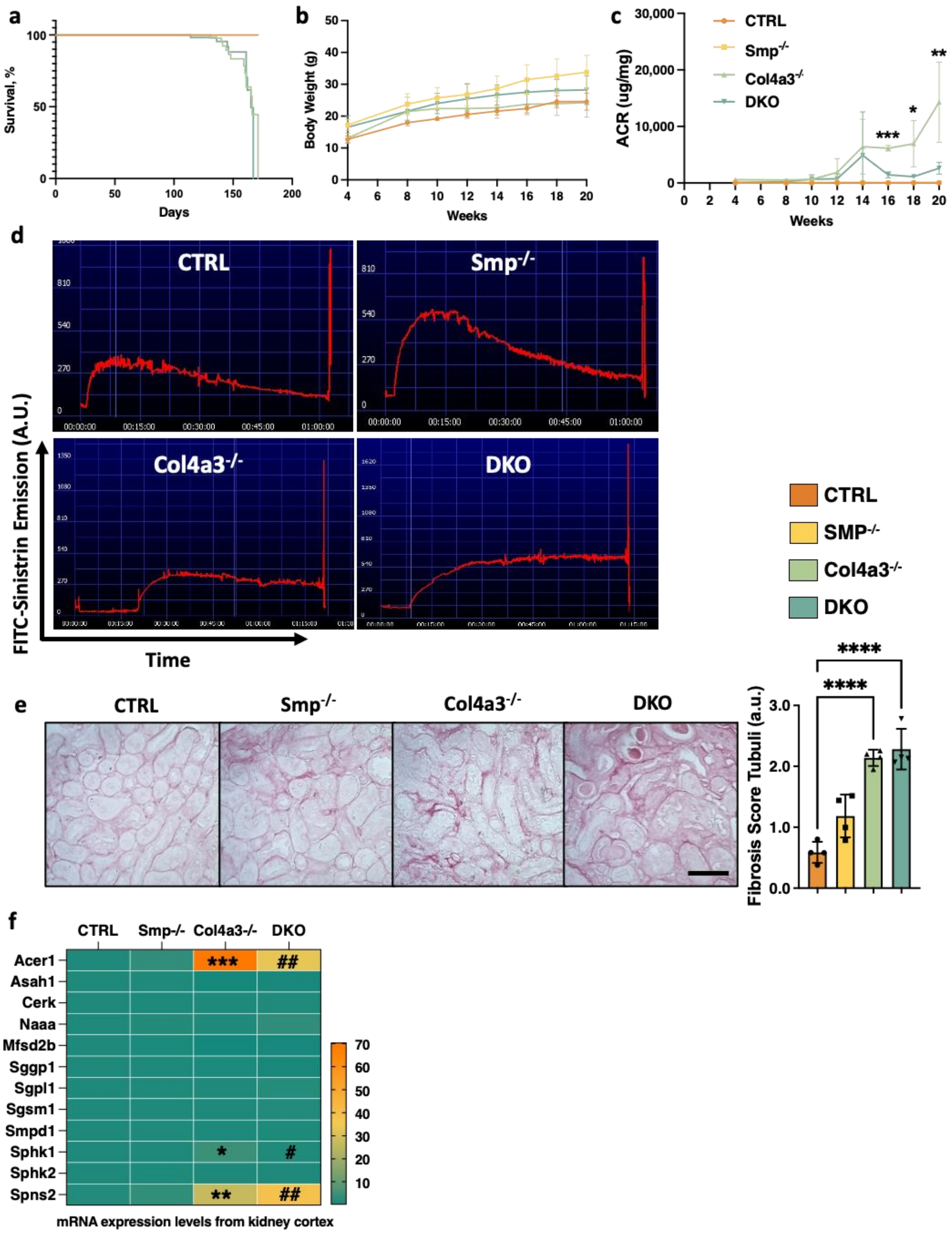

Supplementary Figure S5. Additional phenotypical characteristics of Col4a3$^{-/-}$ mice with podocyte specific *Smpdl3b* deletion. (a) Kaplan-Meier mortality curve showing lifespan in wildtype mice (CTRL, n=10), mice with experimental AS (Col4a3$^{-/-}$, n=14) and Col4a3$^{-/-}$ mice with podocyte specific *Smpdl3b* deletion (DKO, n=14). **(b-c)** Bi-weekly changes in body weight **(b)** and albumin-to-creatinine ratio (ACR) **(c)** in CTRL (n=11), mice with podocyte specific *Smpdl3b* deletion (Smp$^{-/-}$, n=11), Col4a3$^{-/-}$ mice (n=11) and DKO mice (n=10). **(d)** Examples of FITC-sinistrin elimination kinetics curves representative of GFR levels in CTRL, Smp$^{-/-}$, Col4a3$^{-/-}$ and DKO mice. While CTRL and Smp$^{-/-}$ mice show normal FITC-sinistrin elimination, Col4a3$^{-/-}$ and DKO mice show a decreased speed of FITC-sinistrin elimination, indicating reduced GFR. **(e)** Representative picrosirius red staining (x20) in tubules (left panel) and bar graph analysis (right panel) in CTRL (n=4), Smp$^{-/-}$ (n=4), Col4a3$^{-/-}$ (n=4) and DKO

(n=4) mice. **(f)** Heat map showing gene expression changes in mRNA isolated from kidney cortices of 20-week-old CTRL (n=5), Smp$^{-/-}$ (n=5), Col4a3$^{-/-}$ (n=5) and DKO (n=5) mice. Upregulated mRNA expression is represented in orange color and downregulated mRNA expression is represented in green color. Significant changes in the Col4a3$^{-/-}$ compared to the CTRL group are marked with an asterisk (*). Significant changes in DKO compared to Col4a3$^{-/-}$ group are marked with a pound mark (#). Data are mean±SD. *P* values were calculated using One-Way ANOVA.

Abbreviations: Acer1 – alkaline ceramidase 1; Asah1 – N-acylsphingosine aminohydrolase 1; Cerk – ceramide kinase; Naaa – N-acylethanolamine acid amidase; Mfsd2b – MFSD2 lysolipid transporter B, sphingolipid; S1pr1 – S1pr5 – sphingosine-1-phosphate receptors 1-5; Sggp1 – sphingosine-1-phoshate phosphatase; Sgpl1 – sphingosine-1-phosphate lyase; Sgsm1 – sphingomyelin synthase 1; Smpd1 – sphingomyelin phosphodiesterase 1; Sphk1 and Sphk2 – sphingosine kinase 1 and 2; Spns2 – spinster homologue 2.

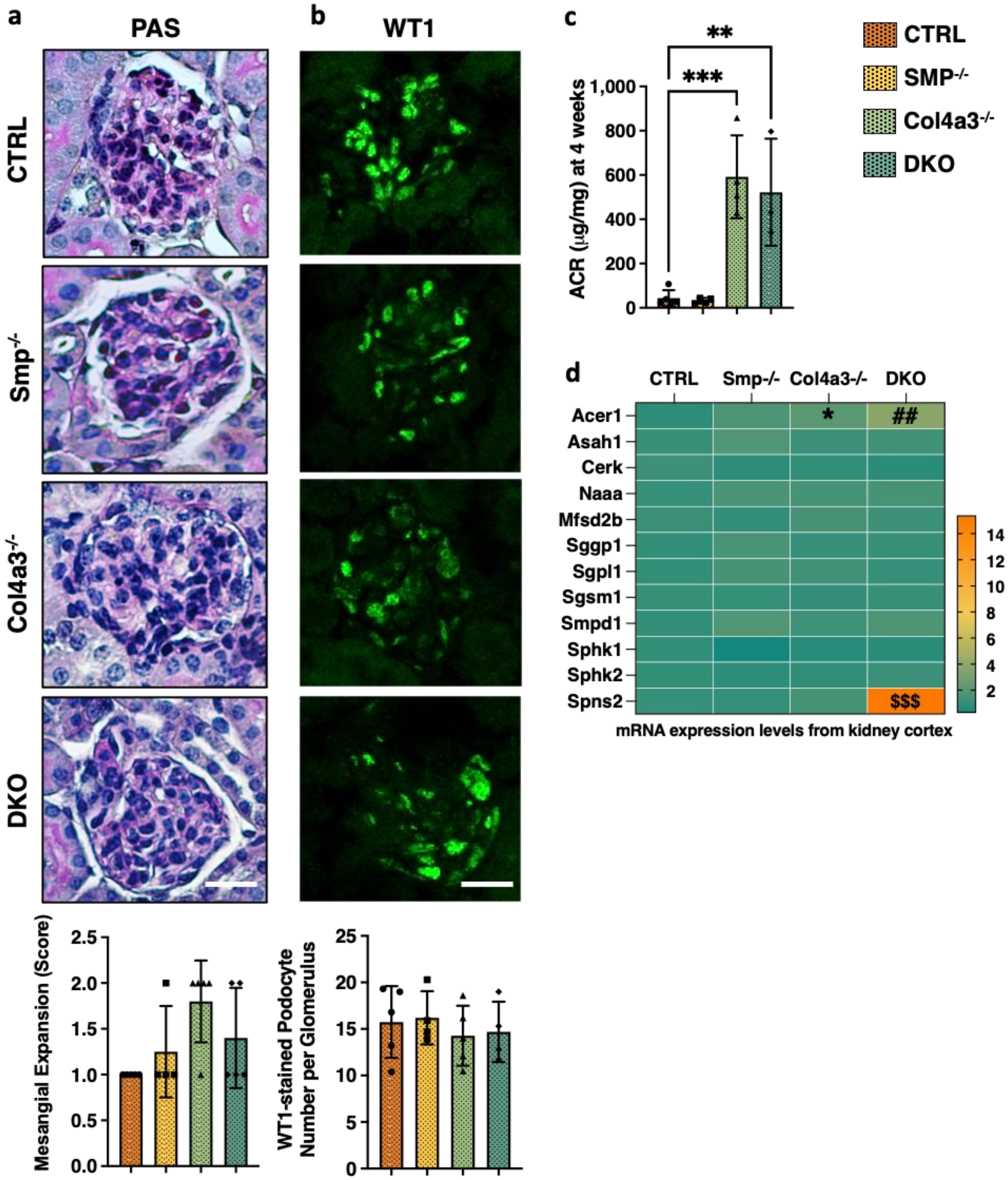

Supplementary Figure S6. Phenotypical characteristics of 4-week-old Col4a3$^{-/-}$ mice with podocyte-specific *Smpdl3b* deletion. (a) Representative PAS staining (x20) 4 μm frozen sections and

bar graph analysis in wildtype mice (CTRL, n=5), mice with podocyte specific *Smpdl3b* deletion (Smp$^{-/-}$, n=5), mice with experimental AS (Col4a3$^{-/-}$, n=5) and Col4a3$^{-/-}$ mice with podocyte specific *Smpdl3b* deletion (DKO, n=5). **(b)** Representative immunostaining (x40) for anti-Wilms' Tumor 1 in 4 μm frozen sections and in CTRL (n=5), Smp$^{-/-}$ (n=5), Col4a3$^{-/-}$ (n=5) and DKO (n=5) mice. **(c)** Albumin-to-creatinine ratio (ACR) in CTRL, Smp$^{-/-}$, Col4a3$^{-/-}$ and DKO groups of mice. ***p<0.001, One-Way ANOVA. **(d)** Heat map showing gene expression changes in mRNA isolated from kidney cortices of 4-week-old CTRL (n=5), Smp$^{-/-}$ (n=5), Col4a3$^{-/-}$ (n=5) and DKO (n=5) mice. Upregulated mRNA expression is represented in orange color and downregulated mRNA expression is represented in green color. Significant changes in the Col4a3$^{-/-}$ compared to the CTRL group are marked with an asterisk (*). Significant changes in DKO compared to Col4a3$^{-/-}$ group are marked with a pound mark (#). Significant changes in DKO compared to CTRL group marked with dollar sign ($). *P* values were calculated using One-Way ANOVA.

Abbreviations: Acer1 – alkaline ceramidase 1; Asah1 – N-acylsphingosine aminohydrolase 1; Cerk – ceramide kinase; Naaa – N-acylethanolamine acid amidase; Mfsd2b – MFSD2 lysolipid transporter B, sphingolipid; S1pr1 – S1pr5 – sphingosine-1-phosphate receptors 1-5; Sggp1 – sphingosine-1-phoshate phosphatase; Sgpl1 – sphingosine-1-phosphate lyase; Sgsm1 – sphingomyelin synthase 1; Smpd1 – sphingomyelin phosphodiesterase 1; Sphk1 and Sphk2 – sphingosine kinase 1 and 2; Spns2 – spinster homologue 2.

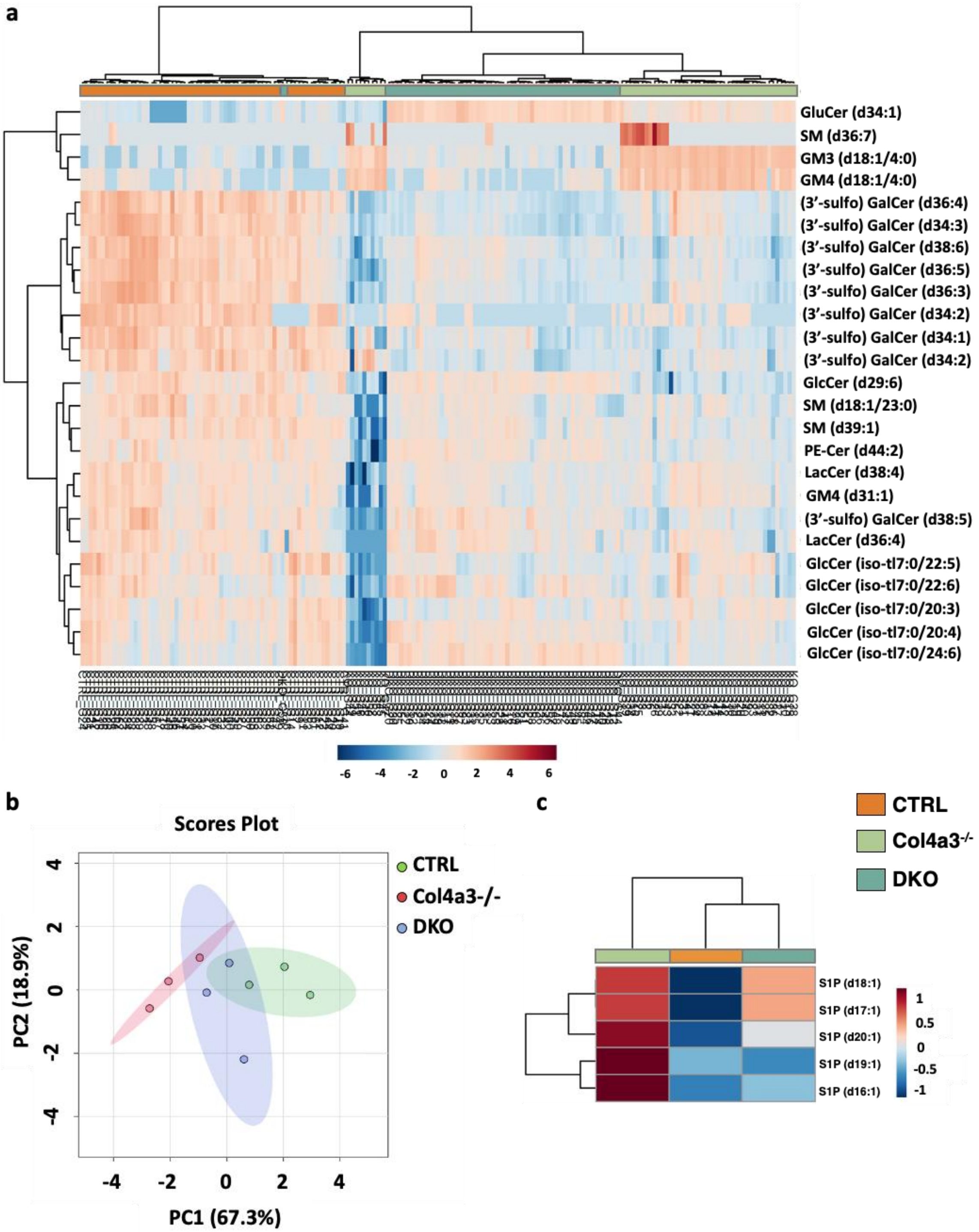

Supplementary Figure S7. Spatial lipidomic analysis of glomerular glycosphingolipid species in Col4a3$^{-/-}$ mice and Col4a3$^{-/-}$ mice with podocyte-specific *Smpdl3b* deficiency. Three experimental groups of mice were used in the study: 1) wildtype control mice (CTRL, n=3), 2) Col4a3$^{-/-}$ (n=3), and 3) Col4a3$^{-/-}$ mice with podocyte specific *Smpdl3b* deletion (DKO, n=3). **(a)** Heatmap of the top 25 annotations with highest Variable Importance in Projection (VIP) scores. Increased levels of the sphingolipid species are highlighted in red and decreased levels of the sphingolipid species are highlighted in blue. **(b)** Unbiased principal component analysis (PCA) of S1P species in CTRL (green), Col4a3$^{-/-}$ (red) and DKO (blue) mice. **(c)** Heatmap of S1P species analysis in the kidney of CTRL, Col4a3$^{-/-}$ and DKO mice. Upregulated S1P species are presented in red color and downregulated S1P species are presented in blue color.

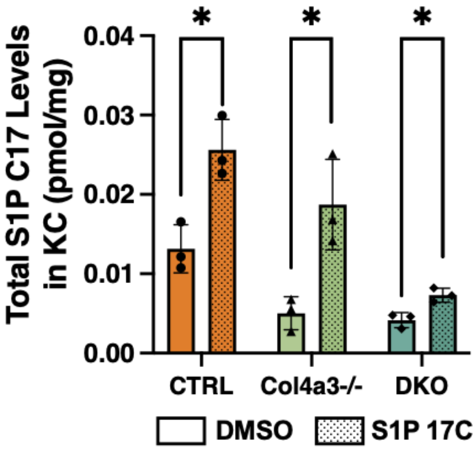

Supplementary Figure S8. LC-MS analysis of S1P C17:0 presence in the kidney. Control mice (CTRL, n=3), mice with the experimental Alport syndrome and normal expression of *Smpdl3b* (Col4a3$^{-/-}$, n=3) and mice with the experimental Alport syndrome and podocyte specific *Smpdl3b* deletion (DKO, n=3) were used to analyze efficacy of S1P intraperitoneal injections and analyze how much S1P C17:0 reached the kidney.

SUPPLEMENTARY TABLES

Supplementary Table S1. List of primers used in the study.

Primer name	Sense 5' → 3'	Antisense 5' → 3'
Smpdl3b	GGGACTACCTCTGCGATTCTC	CCTCTCCTAGACTCTCATTGGG
Acer1	CTGATACAGCACCTGCCTGA	GTGCATGTGGTGGTTCAAAG
Asah1	TGGCATGAATTATTGGCTCA	TCTGCAATTCCCCTCATTTC
Cerk	TCATCCTTATCCGGAAGTGC	AGAGGGTCTGTCCTTGCAGA
Naaa	ATTTGGCCTCTTGACCCTCT	GGGTCTCCAGATTGAGGTGA
Mfsd2b	CCTTAATCGCACTGGCCTACTTCT	CAACCACGGCAGCTGCAATACAAT
Sggp1	TGGGAGCCATTTCCTAGTTG	AGCAAACTTCCCCCAAAACT
Sgpl1	TGGGAGCCATTTCCTAGTTG	AGCAAACTTCCCCCAAAACT
Sgsm1	ACTGGTCACCCAAGAAGGTG	TCTAAGAGTCGCTGCCCATT
Smpd1	TTCCCGAGTGCTGCTTATCT	CCAGGGGTAATCCAGATCCT
Sphk1	GGAGGAGGCAGAGATAAC	TTAGCCCATTCACCACTTCA
Sphk2	GCACGGCGAGTTTGGTTC	GAGACCTCATCCAGAGAGACTAG
Spns2	ATGATGTGCCTGGAATGC	TCAGACTTTCACGGATGCAG
mGapdh	GAAGGGCTCATGACCACAGT	GGATGCAGGGATGATGTTCT
Pod-Cre (genotyping)	CGCACTTCAGTTACTTCAGGTCCTC	GCTTATGCCTGATGTTGATGATGC
LoxP WT (genotyping)	GCACCTGGTTCAGCACTTTG	CAGCTCTGTTCAGCCAGTGA
LoxP KO (genotyping)	TGGCGCAACGCAATTAATGA	AGAACGTCATGGGTTCATCC
Col4a3 WT (genotyping)	TGCTCTCTCAAATGCACCAG	CCAGGCTTAAAGGGAAATCC
Col4a3 KO (genotyping)	GCTATCAGGACATAGCGTTGG	CCAGGCTTAAAGGGAAATCC
pSMP Tg (genotyping)	CCACTTCCTACCCTCGTAAAGTCGAC	GAGGTTGGTCAAGCGTTCCAC
rtTA (genotyping)	CGCACTTCAGTTACTTCAGGTCCTC	GCTTATGCCTGATGTTGATGATGC

Supplementary Table S2. List of antibodies used in the study.

Primary antibodies				
**Antibody and Clone**	**Catalog #**	**Vendor**	**Dilution**	**Application**
Anti-WT1 (C-19)	sc-7385	Santa Cruz Biotech (TX, USA)	1:300	Kidney cortex sections
SMPDL3b	GWB-2281D4	GenWay Biotech (CA, USA)	1:1,000	Murine podocytes; kidney cortex; glomeruli; Immunoperoxidase
Synaptopodin (P-19)	sc-21537k	Santa Cruz Biotech (TX, USA)	1:500	Kidney cortex sections
GAPDH	CB1001-500MG	Millipore (MA, USA)	1:1,000	Murine podocytes; kidney cortex; glomeruli

Secondary antibodies				
Anti-Rabbit (HRP-conjugated)	W401B	Promega Corp. (WI, USA)	1:4,000	Western Blot
Anti-Mouse (HRP-conjugated)	W402B	Promega Corp. (WI, USA)	1:10,000	Western Blot
Anti-Mouse Alexa Fluor 555	A21127	Invitrogen | Thermo Fisher Scientific (CA, USA)	1:500	Immunocytochemistry
Anti-Rabbit Alexa Fluor 488	A11008	Invitrogen | Thermo Fisher Scientific (CA, USA)	1:500	Immunocytochemistry
DAPI	D1306	Invitrogen | Thermo Fisher Scientific (CA, USA)	1:300	Immunocytochemistry

SUPPLEMENTARY DATA

Supplementary Data 1. Pathways enrichment analysis performed using DAVID 6.8. database in Col4a3-/- podocytes, related to Figure 2. An Excel File.

Supplementary Data 2. Lipidomic analysis of sphingolipids in kidney cortices of control (Col4a3+/+) mice and mice with experimental Alport Syndrome (Col4a3-/-), related to Figure 2. An Excel File.

Supplementary Data 3. Lipidomic analysis of sphingolipids in immortalized murine podocytes isolated from control (IMWT) and mice with experimental Alport Syndrome (IMAS), related to Figure 2. An Excel File.

Supplementary Data 4. Lipidomic analysis of sphingolipids in kidney cortices of mice with experimental Alport Syndrome (Col4a3$^{-/-}$) and podocyte-specific *Smpdl3b* deficiency (DKO), related to Figure 3. An Excel File.

Supplementary Data 5. Lipidomic analysis of sphingolipids in urine of mice with experimental Alport syndrome (Col4a3$^{-/-}$) and podocyte-specific Smpdl3b deficiency (DKO), related to Figure 3. An Excel File.

Supplementary Data 6. Lipidomic analysis of sphingolipids in kidney cortex of doxycycline-inducible podocyte specific Smpdl3b overexpression mice, related to Figure 5. An Excel File.

SUPPLEMENTARY REFERENCES

1. Liu X, Ducasa GM, Mallela SK*, et al.* Sterol-O-acyltransferase-1 has a role in kidney disease associated with diabetes and Alport syndrome. *Kidney Int* 2020; **98:** 1275-1285.

2. Saleem MA, O'Hare MJ, Reiser J*, et al.* A conditionally immortalized human podocyte cell line demonstrating nephrin and podocin expression. *J Am Soc Nephrol* 2002; **13:** 630-638.

3. Fornoni A, Sageshima J, Wei C*, et al.* Rituximab targets podocytes in recurrent focal segmental glomerulosclerosis. *Sci Transl Med* 2011; **3:** 85ra46.

4. Granado MH, Gangoiti P, Ouro A*, et al.* Ceramide 1-phosphate (C1P) promotes cell migration Involvement of a specific C1P receptor. *Cell Signal* 2009; **21:** 405-412.

5. Bielawski J, Szulc ZM, Hannun YA*, et al.* Simultaneous quantitative analysis of bioactive sphingolipids by high-performance liquid chromatography-tandem mass spectrometry. *Methods* 2006; **39:** 82-91.

6. Belov ME, Ellis SR, Dilillo M*, et al.* Design and Performance of a Novel Interface for Combined Matrix-Assisted Laser Desorption Ionization at Elevated Pressure and Electrospray Ionization with Orbitrap Mass Spectrometry. *Analytical Chemistry* 2017; **89:** 7493-7501.

7. Trede D, Schiffler S, Becker M*, et al.* Exploring Three-Dimensional Matrix-Assisted Laser Desorption/Ionization Imaging Mass Spectrometry Data: Three-Dimensional Spatial Segmentation of Mouse Kidney. *Analytical Chemistry* 2012; **84:** 6079-6087.

8. Hadi AM, Mouchaers KT, Schalij I*, et al.* Rapid quantification of myocardial fibrosis: a new macro-based automated analysis. *Cell Oncol (Dordr)* 2011; **34:** 343-354.

9. Mitrofanova A, Mallela SK, Ducasa GM*, et al.* SMPDL3b modulates insulin receptor signaling in diabetic kidney disease. *Nature Communications* 2019; **10:** 2692.

10. Shigehara T, Zaragoza C, Kitiyakara C*, et al.* Inducible podocyte-specific gene expression in transgenic mice. *J Am Soc Nephrol* 2003; **14:** 1998-2003.

11. Takahashi N, Boysen G, Li F*, et al.* Tandem mass spectrometry measurements of creatinine in mouse plasma and urine for determining glomerular filtration rate. *Kidney international* 2007; **71:** 266-271.

12. Herrera Pérez Z, Weinfurter S, Gretz N. Transcutaneous Assessment of Renal Function in Conscious Rodents. *J Vis Exp* 2016**:** e53767.

13. Montes GS. Structural biology of the fibres of the collagenous and elastic systems. *Cell biology international* 1996; **20:** 15-27.

14. Crowley SD, Vasievich MP, Ruiz P*, et al.* Glomerular type 1 angiotensin receptors augment kidney injury and inflammation in murine autoimmune nephritis. *The Journal of clinical investigation* 2009; **119:** 943-953.

15. Wei C, El Hindi S, Li J*, et al.* Circulating urokinase receptor as a cause of focal segmental glomerulosclerosis. *Nature medicine* 2011; **17:** 952-960.

16. Ioannou GN, Haigh WG, Thorning D*, et al.* Hepatic cholesterol crystals and crown-like structures distinguish NASH from simple steatosis. *J Lipid Res* 2013; **54:** 1326-1334.

17. Guzman J, Jauregui AN, Merscher-Gomez S*, et al.* Podocyte-specific GLUT4-deficient mice have fewer and larger podocytes and are protected from diabetic nephropathy. *Diabetes* 2014; **63:** 701-714.

18. McCloy RA, Rogers S, Caldon CE*, et al.* Partial inhibition of Cdk1 in G 2 phase overrides the SAC and decouples mitotic events. *Cell Cycle* 2014; **13:** 1400-1412.

19. Charan J, Kantharia ND. How to calculate sample size in animal studies? *J Pharmacol Pharmacother* 2013; **4:** 303-306.
